# Cultural universality and specificity of teacher-student relationship: a qualitative study in Belgian, Chinese, and Italian primary school teachers

**DOI:** 10.3389/fpsyg.2023.1287511

**Published:** 2023-11-14

**Authors:** Canmei Xu, Mariëtte Huizinga, Giuseppe De Luca, Sophie Pollé, Ruwen Liang, Simona Sankalaite, Debora L. Roorda, Dieter Baeyens

**Affiliations:** ^1^Parenting and Special Education Research Unit, Faculty of Psychology and Educational Sciences, Katholieke Universiteit Leuven, Leuven, Belgium; ^2^Department of Education and Family Studies, Faculty of Behavioural and Movement Sciences, Vrije Universiteit Amsterdam, Amsterdam, Netherlands; ^3^Department of Psychology, Alma Mater Studiorum, University of Bologna, Bologna, Italy; ^4^Research Institute of Child Development and Education, University of Amsterdam, Amsterdam, Netherlands

**Keywords:** teacher-student relationship, cross-cultural investigation, qualitative study, metatheme analysis, primary school teacher

## Abstract

Extensive evidence highlights the significant influence of dyadic, emotional teacher-student relationship (TSR) on students’ cognitive functioning, socio-emotional development, and overall well-being. However, it remains unclear whether the TSR construct and its manifestations can be generalized across cultures. This qualitative study investigated TSR among 60 primary school teachers in Belgium, China, and Italy (i.e., countries with varying positions on the collectivistic-individualistic continuum of culture). Through semi-structured interviews and metatheme analysis, the study examined the similarities and differences in TSR across these countries, revealing a nuanced and diverse picture in various cultural contexts. The findings align with the existing TSR model by including dimensions of closeness, conflict, and dependency, while also extending the model to identify additional dimensions such as authority, balance, distance, fairness, increasing student motivation, patience, and strictness. Regarding cultural perspective, teachers from these three countries exhibited similar conceptualizations of closeness, conflict, fairness, increasing student motivation, patience, and strictness, whereas the conceptualization of dependency, authority, balance, and distance may be influenced by (collectivistic versus individualistic) culture. Moreover, the manifestations of TSR varied across countries, highlighting the influence of cultural factors such as cultural norms, collectivistic versus individualistic values, and the perceived legitimacy of teacher authority. These findings shed light on the complexities of TSR across countries and emphasize the significance of culturally sensitive approaches in fostering positive TSR in education.

## 1. Introduction

Ample evidence has been found to support the profound influence of dyadic, affective teacher-student relationship (TSR) on students’ academic performance, socio-emotional and cognitive functioning, as well as their general wellbeing (Sabol and Pianta, 2012; McGrath and Van Bergen, 2015; Lei et al., 2016; Vandenbroucke et al., 2017; Roorda et al., 2021). Building on everyday teaching practices, researchers strongly suggest improving the quality of TSR in the classroom in order to optimize children’s cognitive functioning development and social adjustment (for review, see Kincade et al., 2020). Despite the importance of TSR, the current understanding of the TSR construct and the manifestation (or expressions) of these relationships in teaching practices is predominantly based on a Western individualistic perspective. Some previous cross-sectional studies based on questionnaire data have indicated that students and teachers from individualistic countries perceive their relationships differently compared to those from collectivistic countries (Jia et al., 2009; Yang et al., 2013; Chen et al., 2019, 2023). However, these studies have yielded inconsistent findings (Jia et al., 2009; Yang et al., 2013; Chen et al., 2019, 2023). Furthermore, TSR measurement demonstrated only partially strong invariance between students between cultures, for instance in studies comparing Chinese and Dutch samples (Chen et al., 2019, 2023). This suggests commonalities and variations in the TSR measurement, prompting further exploration of cultural similarities and differences in the TSR concept and manifestation. The current study, therefore, delves into the cultural differences and similarities of how TSR is expressed and understood in greater detail. More specifically, through semi-structured interviews, this study aims to explore the conception of TSR by teachers from Belgium (typically individualistic culture across its different regions), China (typically collectivistic culture across its different regions), and Italy (coexistence of individualistic and collectivistic orientations for northern and southern regions, respectively) (Iacovu, 2002; Hofstede et al., 2010; Heu et al., 2019; Lo Cricchio et al., 2019; Burton et al., 2021).

## 2. Literature review

### 2.1. Teacher-student relationship: concept and manifestation

Dyadic, affective TSR refers to the emotional and social connection between a teacher and an individual child, which develops through moment-to-moment interactions (Pianta, 1999). Attachment theory suggests that TSR, as a form of adult-child relationships in the school context, can provide children with new attachment experiences that help them to reframe their previous attachment models of self and others. TSR acts as a compensatory resource for children, particularly those with insecure or negative parent-child relationships (Sabol and Pianta, 2012; Verschueren, 2015; Vandenbroucke et al., 2017). High-quality TSR has been found to predict improvements in cognitive performance (e.g., de Wilde et al., 2016; Cadima et al., 2019) and socio-emotional functioning (e.g., O’Connor et al., 2011; Camodeca and Coppola, 2019). In contrast, negative TSR, such as a high degree of conflict and dependency, is associated with more externalizing and internalizing problems in children (e.g., Lei et al., 2016; Roorda et al., 2021). According to attachment theory, adults whom a child has bonded with, such as parents or teachers, play a dual role of “secure base” and “safe haven” for the child (Bowlby, 1969). First, by providing a secure base, teachers enable children to explore new experiences and engage more in classroom activities according to teachers’ instructions. Additionally, teachers can serve as a safe haven for children, providing them with comfort, safety, and reassurance when they experience fear or stress. This support can play a pivotal role in helping children regulate their stress levels and foster their cognitive and socio-emotional development (Verschueren and Koomen, 2012).

TSR has been conceptualized into three dimensions: closeness, conflict, and dependency (e.g., Pianta, 2001). Closeness is characterized by open communication, warmth, and affection between teachers and children, with teachers serving as both a secure base and a safe haven. For example, when children feel close to their teachers, they openly share their feelings and experiences. On the other hand, if children do not see their teachers as secure bases, they might become overly dependent on them and be reluctant to explore independently, leading to dependency issues. For instance, children might react strongly to separation from their teachers or want to stay in close proximity to them all the time. Conflict arises when children do not seek comfort from their teachers because they do not see them as a safe haven, and have negative feelings toward the teacher. In other words, conflict measures the level of fights, negativity, and disharmony in the relationship. Previous meta-analyses have shown that teacher-student closeness is related to improved school engagement and academic achievement, as well as fewer externalizing problems in children. On the other hand, teacher-student conflict and dependency are associated with lower school engagement, academic achievement, and higher levels of internalizing and externalizing problems (Lei et al., 2016; Roorda et al., 2017, 2021). Therefore, a comprehensive understanding of these dimensions of TSR is crucial for cultivating positive relationships between teachers and students, preventing negative interactions, and nurturing the growth of children.

### 2.2. Teacher-student relationship and culture

Despite numerous studies on TSR (especially in individualistic cultures), it is unclear whether the definitions and descriptions of TSR are universally accepted and applied. The cultural context shapes how people perceive and interact with the world, including their self-concept and their relationships with others (Han and Mäkelä, 2020). Individualistic cultures emphasize independence, autonomy, and egalitarian relationships, while collectivistic cultures value social relatedness, harmony, and hierarchical relationships (Rothbaum et al., 2000; Hofstede et al., 2010; Keller, 2021). For example, during fMRI scans, East Asians exhibited activation in the same brain region (medial prefrontal cortex) when making trait judgments about themselves and their mothers, whereas North Americans and Australians did not show this pattern (Zhu et al., 2007). This finding indicates that culture shapes the structural organization of how individuals perceive themselves and their connections with others.

In terms of TSR, Chen et al. (2019, 2023) found that TSR measurements, such as (teacher-perceived) Student-Teacher Relationship Scale (STRS; Pianta, 2001), Student Perception of Affective Relationship with Teacher Scale (SPARTS; Koomen and Jellesma, 2015), demonstrated only partially strong invariance between students from Netherlands and China. This suggests that certain items related to TSR on these scales may be interpreted differently in individualistic and collectivistic cultures. For instance, when completing the STRS (the short version; Koomen et al., 2012), Chen et al. (2019) discovered that Items 9 and 12 had higher intercept values in the Dutch group. This suggests that Dutch teachers tended to score higher on these items compared to Chinese teachers, when their level of closeness was the same. On the other hand, item 15 had a higher intercept in the Chinese group, indicating that Chinese teachers scored higher on this item than their Dutch counterparts when their level of closeness was equal. Regarding conflict, the intercept values of Items 7 and 11 also differed between Chinese and Dutch teachers. This might indicate that the manifestation of a secure-based relationship may vary depending on the cultural context. Relatedly, Cheah et al. (2015) found that, in individualistic cultures, the caregiver’s warmth or closeness is expressed through direct and outward demonstrations such as verbal expressions, hugging, and kissing. Collectivistic cultures, however, tend to demonstrate warmth and closeness through physical nurturance, such as preparing the child’s preferred meals and providing proper educational guidance. In addition, regarding caregivers or teachers exerting pressure and promoting conformity, although this behavior could be perceived as expressions of love, care, and culturally accepted practices within collectivistic cultures, such approaches might be considered maladaptive in individualistic cultures, which could potentially lead children to experience feelings of control or distress (Deci and Ryan, 1987; Zhou et al., 2012; Louie et al., 2013).

Conflict-related emotions are also expressed differently across cultures. In individualistic cultures, expressing hostile feelings is essential for communicating emotions and needs and maintaining social bonds (Srivastava et al., 2009), which is encouraged for secure attachment (Bowlby, 1969). Conversely, in collectivistic cultures, emotional openness is seen as an irrational impulse and useless (Chiang, 2012). Instead, these cultures prioritize emotional self-control and restraint, valuing emotional suppression as a strategy for maintaining interpersonal harmony (Rothbaum et al., 2000; Han, 2017). For instance, Wei et al. (2013) found a significant positive association between emotional suppression and interpersonal harmony among Chinese-American students, but no such association for European-American students, suggesting that emotional suppression is perceived as a more positive quality among collectivistic rather than individualistic students.

The current understanding of dependency, admittedly, also primarily stems from Western individualistic cultures, emphasizing children’s autonomy and exploration in the presence of a teacher (autonomy-seeking; Strand, 2020). In these cultures, a child’s reluctance to explore independently (dependency) is viewed as an insecure attachment associated with negative outcomes, such as reduced achievement and prosocial behavior, as well as increased externalizing behaviors (Roorda et al., 2021). However, this conceptualization may not universally apply to collectivistic cultures. In collectivistic cultures, parents and teachers value strong social bonds within social groups (security-seeking; Strand, 2020), and may consider dependency as an element in fostering closeness with the child (Gregoriadis and Tsigilis, 2008; Gregoriadis et al., 2021). For example, studies in countries with individualistic values, such as the United States of America, have revealed a non-significant to negatively significant correlation between dependency and closeness (Webb and Neuharth-Pritchett, 2011). However, students in more collectivistic countries, such as Greece and Iran, indicate a significant positive association between these two concepts (Tsigilis et al., 2018; Gregoriadis et al., 2021; Vahidi et al., 2022). Despite these past quantitative cross-cultural investigations, it is still unclear whether teaching practices related to TSR are culturally universal or specific. Therefore, a qualitative cross-cultural analysis of TSR conceptualization and manifestation is warranted in the current study.

### 2.3. The current study

Semi-structured interviews were conducted with first-to-third teachers from Belgium, China, and Italy to explore TSR’s cultural conceptualization and manifestation in teaching practices. The semi-structured interview serves as a valuable narrative method designed to evaluate teachers’ implicit mental representations of dyadic TSR (Koenen et al., 2019; Kennedy et al., 2022). By leveraging narrative data, this study can illuminate the unconscious cognitive processes at play among teachers (Maier et al., 2004) and provide a deeper understanding of how TSR is manifested and imbued with cultural significance. Moreover, previous research has indicated that TSR measurement demonstrated only partially strong invariance between students from Netherlands and China (Chen et al., 2019, 2023), potentially limiting the scale’s ability to fully capture the concept and additional items. Thus, the current study can also complement prior cross-cultural research on TSR by advancing the development of culturally sensitive measurements for TSR.

## 3. Methods

### 3.1. Participants

The interviews of the present study employ the STRS framework (Pianta, 2001), which is suitable for teachers working with students from preschool through grade 3. Given the significant impact of TSR in early formal education on students’ long-term outcomes (Lei et al., 2016; Ansari et al., 2020), this study focuses on teachers engaged with first- to third-grade students. This included 20 interviews with Belgian teachers, 20 with Chinese teachers, and 20 with Italian teachers, as previous research indicated that 16 to 24 interviews in certain contexts were necessary to reach saturation (i.e., the point that all relevant conceptual categories were thoroughly explored and exhausted; Hennink et al., 2017). In the present study, code saturation was achieved with 17 interviews in each country, as indicated by the identification of more than 95% of the codes (Hennink et al., 2017). Different from previous cross-cultural studies, the current study compared the interviews of teachers from three different countries: Belgium is known for its typical individualistic culture that values the autonomy of students (Coşkan et al., 2016), while China, as a highly collectivistic society, emphasizes common goals and relatedness among students (Haberstroh et al., 2002). Italy has been recognized as an individualistic society but has maintained its collectivistic nature, particularly in the southern regions (Heu et al., 2019; Lo Cricchio et al., 2019; Burton et al., 2021). In the current study, teachers from northern and southern Italy were recruited. Teachers with at least 2 years of teaching experience were recruited to ensure the teachers’ expertise in understanding the complexities of the TSR within their cultural contexts (Alban-Metcalfe et al., 2002). Participant demographics are presented in Table 1. To protect participant anonymity and privacy, pseudonyms have been used.

**TABLE 1:** Sociodemographics of the interviewed teachers in Belgium, China, and Italy.

Pseudonymized participant	Country	Urban/ rural	Gender	Teaching experience (years)	Grade	Subject
Antje	Belgium	Rural	Female	7	1st, 3rd, and 5th	Languages, math, spelling, etc.
Michiel	Belgium	Rural	Male	6	1st	All subjects except physical education
Marselien	Belgium	Rural	Female	33	1st through 3rd	Support for different subjects, especially math and Dutch
Febe	Belgium	Rural	Female	32–33	1st and 2nd	Art, languages, math, world orientation, geography, history, etc.
Catho	Belgium	Rural	Female	16	3rd	Main subjects including religion but not world orientation or physical education
Krista	Belgium	Rural	Female	8+	1st	All subjects except physical education and information and communications technology (ICT)
Fien	Belgium	Rural	Female	4	1st	Languages, math, religion, world orientation, music, social skills, writing
Dora	Belgium	Rural	Female	8	1st	All subjects
Charlotte	Belgium	Rural	Female	34	2nd	All subjects except presentation skills and music
Mirte	Belgium	Rural	Female	30+	2nd	All subjects
Hanne	Belgium	Urban	Female	6	2nd	Dutch, world orientation, social skills, geometry, writing
Emma	Belgium	Rural	Female	9	1st	Everything, except physical education and science, technology, engineering en mathematics (STEM)
Sara	Belgium	Rural	Female	35	1st	Everything, except physical education and religious studies
Femke	Belgium	Rural	Female	8	2nd	Everything, except physical education and world orientation
Kato	Belgium	Rural	Female	4	3rd	Everything, except physical education
Anke	Belgium	Urban	Female	9	2nd	Everything, except physical education
Lieze	Belgium	Rural	Female	6	1st	World orientation, geometry, Dutch, religious studies, music
Joke	Belgium	Rural	Female	39	1st	Everything, except physical education
Nina	Belgium	Rural	Female	6	2nd	Everything, except physical education
Ellen	Belgium	Urban	Female	13	3rd and 4nd	Everything, except physical education
Jiahui	China	Urban	Male	2	1st and 3rd	Physical education
Biling	China	Rural	Female	24	1st	Chinese
Shiliu	China	Rural	Female	27	1st	Chinese
Kaiwen	China	Urban	Male	8	1st through 6th	Physical education
Bohan	China	Rural	Male	4	2nd	Math
Caibai	China	Urban	Female	34	1st through 3rd	Chinese
Mumei	China	Urban	Female	7	3rd	Art
Yuean	China	Urban	Female	2.5	2nd	Chinese
Dongling	China	Rural	Female	4	2nd and 3rd	Chinese and English
Zian	China	Rural	Female	5	1st through 3rd	Chinese
Yuxue	China	Urban	Female	5	2nd	Chinese
Wanning	China	Urban	Female	30	1st through 6th	Chinese
Yizhen	China	Rural	Female	6	1st and 2nd	Art and Math
Anhe	China	Urban	Female	8	1st through 3rd	Music
Jingzhu	China	Urban	Female	6	1st through 6th	Music
Meixi	China	Rural	Female	4	1st through 6th	Music
Xinqi	China	Rural	Female	15	1st through 3rd	Math
Taizhe	China	Urban	Male	17	1st through 6th	Psychological education
Yuanyuan	China	Urban	Female	15	2nd	Chinese
Lvxia	China	Rural	Female	7	1st through 3rd	Chinese
Ace	Italy	Rural	Female	4	2nd	Math, history, geography, science, art, technology, music
Debbie	Italy	Urban	Female	12	3rd	Italian
Rendy	Italy	Rural	Female	35	3rd	Italian, art
Buddy	Italy	Rural	Female	16	2nd	Math, English, science, technology, computer science
Grace	Italy	Rural	Female	5	3rd	Italian, history, English, art
Heidi	Italy	Rural	Female	33	2nd	Math, science, English, history, geography, civic education, physical education, technology
Rennah	Italy	Urban	Female	6	3rd	Italian, Math, history, geography, computer science, civic education, science, art, music
Aisha	Italy	Rural	Female	15	1st	Math, science, technology, physical education
Chynthia	Italy	Urban	Female	20	3rd	Math
Paula	Italy	Urban	Female	14	1st	Math, science, technology, physical education, music
Nunzia	Italy	Rural	Female	6	3rd	Italian, history
Boldy	Italy	Rural	Male	3	3rd	Italian, Math, science
Gerlanda	Italy	Rural	Female	19	3rd	Italian, Math, science, geography, art, physical education
Leoluca	Italy	Rural	Female	18	1st	Italian, Math, history, geography, computer science, technology, civic education, science, art, music
Palma	Italy	Rural	Female	22	2nd and 3rd	Math, English
Smily	Italy	Rural	Female	34	3rd	History, geography, music, physical education
Onofria	Italy	Rural	Female	18	1st	Italian, English, science, technology, music, art
Amanda	Italy	Rural	Female	5	2nd and 3rd	Math, science, geography, English, physical education, music, art, technology
Billie	Italy	Rural	Female	15	1st	Italian, history, geography, Math, English, art, music

### 3.2. Procedure and measurements

This study was approved by the Social and Societal Ethics Committee of Katholieke Universiteit Leuven. All participants gave their informed consent prior to the interviews. Trained and native interviewers conducted interviews with participating teachers in their mother tongue (i.e., Dutch, Mandarin, and Italian for the Belgian, Chinese, and Italian participants, respectively) in each country. The interviews had a duration of approximately 40 to 60 min each and were audio-recorded and transcribed for coding. The questioning sequence was standardized and presented in Table 2, with all participating teachers in the three countries being asked the same set of questions in the same order. The first question (Q1) asked teachers to describe their general relationships with their students in their own terms, based on their personal experiences and understanding and definition of TSR. This open-ended question aimed to discover how teachers from different countries think about relationships with students, without being prompted in any specific way. In the following three questions (Q2 to Q4), teachers were provided with the definitions of closeness, conflict, and dependency, respectively, based on the theoretical framework of TSR quality by Pianta (2001). Teachers were then asked to specify which behaviors they thought were typical for such kinds of relationships and to provide some real-life experiences. These questions aimed to uncover how teachers from different countries interpreted (the manifestations of) these three relationship dimensions. Finally, an open-ended question (Q5) was included to explore any additional relationship themes that may have emerged. Upon completion of the interviews, participating teachers were compensated with a 10-euro gift voucher.

**TABLE 2:** Interviewed questions.

Questions
Q1. Can you broadly describe the relationship between you and your pupils? Can you elaborate a bit more on that?; (if they could recall nothing, invite them to use three adjectives to describe) Q2. If you define closeness as that the teacher and a child experiencing warmth, positive emotions, and having the feeling that they can talk freely with each other about important things, what behaviors could be regarded as closeness between children and the teacher? Could you give me some real-life examples and describe them as specific as possible? Q3. If you define conflict as the degree to which the teacher perceives his/her relationship with a child as full of negative feelings, anger, having fights or struggles with each other regularly, and the child having the idea that he/she is treated unfairly by the teacher, what behaviors could be regarded as conflict between children and the teacher? Could you give me some real-life examples and describe them as specific as possible? Q4. If you define dependency as the degree to which a student asks for help when not really needed, responds strongly to separation from the teacher and wants to be close to the teacher all the time, what behaviors could be regarded as dependency between the child and the teacher? Could you give me some real-life examples and describe them as specific as possible? Q5. Apart from the things we discussed so far, are there any other things that you think are also important when we talk about relationships with students? Could you tell me more about it?

## 4. Analyses

Two independent coders per country were involved throughout the analyses. Prior to the analysis process, all the coders conducted a cultural reflection. The reflection involved considering how the cultural background of the researchers may influence the research process, shaping each interview and analytical decision that was made before coding began, which is presented in Supplementary Appendix 1. This step was taken to minimize cultural bias during the analysis process.

**FIGURE 1:**
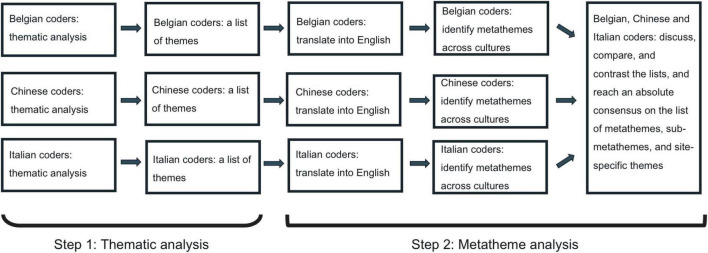
Process model for team-based metatheme analysis in the current study.

To identify the cultural universality and specificity of TSR, coders followed the metatheme analysis presented in Figure 1, which draws upon established procedures for thematic analysis adapted for cross-cultural ethnography and qualitative research (Wutich et al., 2021). For instance, Trainer et al. (2022) applied metatheme analysis to explore themes related to blame and feelings of responsibility concerning weight and “fat” across four cultural groups. This approach combined two steps, enabling us to discover site-specific themes (i.e., themes that teachers mentioned within a specific country), sub-metathemes (i.e., themes that cut across at least two countries), and metathemes (i.e., broader themes based on the sub-metathemes). Site-specific themes exclusive to one country will be retained as is.

### 4.1. Step 1 of the metatheme analysis

In Step 1 of the metatheme analysis, site-specific theme identification was conducted using thematic analysis. After transcribing all teacher interviews, each group of coders determined the site-specific themes in the language of data collection: Dutch in Belgium, Mandarin in China, and Italian in Italy. As a foundational method for qualitative analysis, thematic analysis probes how individuals perceive and interpret their own experiences in a particular context (Braun and Clarke, 2006). To accomplish this, coders followed the widely used approach developed by Braun and Clarke (2006) by taking seven steps to perform the thematic analysis. Two rounds of analyses were performed by adopting an inductive and a deductive approach to the TSR concept. In the first round, coders read the transcripts related to Q1 and Q5, and created a theme list for “the concepts of general teacher-student relationship” for each country. In the second round, coders generated the list for “the manifestation of teacher-student closeness, conflict, and dependency (i.e., the pre-defined dimensions of TSR)” based on Q2, Q3, and Q4. The coders discussed the codes and validated each code list within the country. The inter-rater reliability was determined through a coding comparison in NVivo (Jackson and Bazeley, 2019), where one coder analyzed all interviews, and a second coder reviewed 20% of the interviews to establish an agreement between the two coders, using characters as a basis for calculation. The inter-rater reliability was satisfying: for the first round, Kappa was 0.85, 0.86, 0.93 for Belgium, China, and Italy, respectively; for the second round, Kappa was 0.95, 0.89, 0.80 for Belgium, China, and Italy (Burla et al., 2008; MacPhail et al., 2016).

### 4.2. Step 2 of the metatheme analysis

In Step 2 of the metatheme analysis, one coder of each country carefully translated the theme list and corresponding quotes into English while ensuring that the original meanings were preserved. For example, in the Dutch-speaking group, one teacher used the phrase “Maar het is niet dat ze aan mij hangen” (literally, “But it is not the case that they hang on my leg”) to describe the intensity of the child’s affectionate behavior toward the teacher. Next, to compare and merge metathemes related to the conceptualization and manifestation of TSR (including closeness, conflict, dependency, and potential other dimensions), the coders conducted a collaborative sorting activity (Wutich et al., 2021; Trainer et al., 2022). Within each country, the coders identified common or specific themes across the lists of themes and transcripts from all groups. For instance, Belgian coders coded interviews from all three cultural contexts (Belgian, Chinese, and Italian), and collaborated to develop a list of metathemes, sub-metathemes, and site-specific themes. The same process was followed by Chinese and Italian coders. To ensure the accuracy and nuance of the metathemes, the coders discussed and refined the definitions of each metatheme by drawing on quotes from the interviews.

When the coders generated the lists of metathemes, sub-metathemes, and site-specific themes for each country, they thoroughly discussed areas of convergence and divergence. During this discussion, the coders clarified their understanding of each metatheme and identified any potential overlaps or contradictions. For example, one theme that initially emerged was “coolness,” which was included as a part of the metatheme “conflict.” However, during the discussion, the coders realized that “coolness” reflects more neutrally detached relationships, containing unique meanings distinct from the metatheme of “conflict,” encompassing themes related to negative feelings and struggling relationships between teachers and students. As a result, the coders incorporated the “coolness” theme into the larger metatheme of “distance,” refining the definition and examples accordingly.

## 5. Results

Table 3 illustrates the results of Round 1 and Round 2 analyses, presenting the identified four metathemes and 48 sub-metathemes. The table also emphasizes the level of variability within each sub-metatheme across study samples, with culturally specific sub-metathemes indicated in bold text. The comprehensive list of metathemes, sub-metathemes, and specific themes for each country is available in Supplementary Appendices 2–5. In what follows, all metathemes and sub-metathemes will be briefly introduced to facilitate a clear understanding of what each metatheme represents. Following that, all the sub-metathemes will be elaborated upon by presenting data related to the 31 culturally universal and 17 culturally specific sub-metathemes, accompanied by some illustrative quotes using pseudonyms.

**TABLE 3:** Summary of metathemes across Belgian, Chinese and Italian group.

Cross-site metatheme	Sub-metatheme (definition)	Sites-specific themes (1st round)			Sites-specific themes (2nd round)		
		Belgium	China	Italy	Belgium	China	Italy
Closeness	Acceptance by teachers (Students feeling understood by the teacher and the possible actions that can cause this)	1	0	1	1	1	1
	Actively looks for contact (The teacher/student initiates communication)	0	0	0	0	1	1
	Affection (Student/teacher verbally or physically express that he/she likes the teacher/student)	1	1	1	1	1	1
	**Attunement** (The teacher is in tune with what the student is feeling)	0	0	0	1	1	1
	**Cares about the student** (Teacher taking into account the emotions, physical health and wellbeing of students)	1	1	1	1	1	1
	Cooperation (Teacher and student working together smoothly, or student follows teacher’s suggestions readily)	1	1	1	1	1	1
	**Gift-giving** (Teacher and student give gifts to each other)	0	1	1	1	1	1
	**Greetings** (Greetings between teacher and student in daily life)	0	1	0	0	1	1
	**Having an active interest in each other’s life** (Every attempt of the teacher/student to get to know/understand the student/teacher)	0	0	0	1	0	1
	Having fun together (Every act of teacher and students enjoying each other’s company and doing fun activities)	1	1	1	1	1	1
	**Interest in lessons** (The student is interested in what teacher teaches)	0	1	0	0	1	1
	Keeping track of the teacher (The student keeps track of the teacher or expects presence of the teacher)	0	0	0	1	1	1
	Looking up to the teacher (Teacher as a point of reference for students and in the position to model certain behaviors)	1	1	0	1	0	1
	Open communication (Every act of the teacher to open a channel of communication towards students and students sharing personal experiences/opinions)	1	1	1	1	1	1
	**Regarding as a member of the family/friend** (Student/teacher regards the teacher/student as his or her family/friend)	0	1	0	1	1	1
	Respect (Teachers and students respect each other and/or the rules)	1	1	1	1	1	1
	Safe haven (The student seeks comfort and reassurance from the teacher)	0	0	0	1	1	1
	Satisfactory relationship (The student/teacher has positive feelings about relationship with teacher/student)	0	0	0	1	1	1
	Secure base (Student feels a sense of safety in their relationship with the teacher)	1	1	1	1	1	1
	Seeking practical help (The student feels free to seek help from the teacher or the teacher is available to help student when needed)	1	0	1	1	1	1
	Trust (Student and teacher having faith in one another)	1	1	1	1	1	1
Conflict	**Conflict resolution** (The teacher/student solves a conflict)	0	0	0	1	1	1
	Dissatisfaction and negative feelings (The teacher/student not being content about his/her teacher/student, or the teacher/student implicitly expresses feelings of anger, hostility or disapproval towards the student/teacher)	1	1	1	1	1	1
	Doubting (Students don’t trust teachers)	1	1	1	0	1	0
	**Fear** (The student is afraid of teacher because the teacher is harsh or strict)	0	0	0	0	1	1
	Non-communication (The student/teacher does not to communicate with the teacher/student)	0	0	0	1	1	1
	**Non-conflict** (The teacher perceives no conflict with the student)	0	0	0	1	1	1
	**Non-cooperative behaviors** (Students’ actions or behaviors that are not collaborative, cooperative, or helpful in achieving a goal or task)	0	0	0	1	1	1
	**Punishment** (The teacher uses corporal punishment)	0	0	0	0	1	1
	Reproachments (The teacher reproaches students)	0	1	0	1	1	1
	Struggling relationship (The teacher feels struggled with the student)	1	1	1	1	1	1
	Unfairness (The teacher treats the student unfairly)	0	0	0	1	1	1
Dependency	Asking for help constantly (The student keeps looking for help, even when it is not really needed)	0	0	0	1	1	1
	Confirmation seeking (The student looks for confirmation by the teacher)	0	0	0	1	1	1
	Demanding attention (The student demands attention or teacher improves students’ getting attention)	0	0	0	1	1	1
	**Emotional dependency** (Chronically and excessively seeking proximity and support)	0	0	0	1	1	1
	Encourage students’ independence (The teacher tries to make students more independent, and/or students are happy being independent)	1	1	1	1	0	1
	Instrumental dependency (Support and help seeking in effective ways)	0	0	0	1	1	1
	**Non-overreliance** (The teacher does not perceive the student’s dependency)	0	0	0	0	1	1
	Physical closeness (inappropriately) (The student needs to be constantly, excessively, or inappropriately physically closed to the teacher)	0	0	0	1	1	1
	**Teacher improves students’ dependency** (Teacher’s behaviors in order to not make students independent)	0	0	0	0	0	1
Other dimensions	**Authority** (Teacher acts as an authoritative figure)	1	1	1	0	1	1
	**Balance** (Teacher needs to keep balance in the classroom)	1	0	0	1	0	0
	**Distance** (The coolness or unfriendliness in the way that teacher/student behaves toward each other)	0	0	0	1	1	1
	Fairness (Teacher treats students in a fair way)	0	0	0	1	1	1
	Increasing student motivation (Teacher improves students)	0	1	1	1	1	1
	Patience (Teacher stays calm and does not get annoyed by the student)	1	1	1	1	0	1
	Strictness (Teacher regards many actions of students as unacceptable and do not allow them)	1	1	1	1	1	1

1(0) represents that each theme was (not) mentioned in the first round interview and analysis (Q1 and Q5) and the second round (Q2, Q3, Q4). Bold text refers to culturally specific themes, while the other themes were identified as culturally universal across the participants.

### 5.1. Metathemes: closeness, conflict, dependency, and other dimensions

Based on the model of Pianta (2001), most of the participants’ descriptions of their relationship with the student generally could be put in the three main metathemes: closeness (including 21 sub-meta-themes), conflict (including 11 sub-metathemes), dependency (including nine sub-metathemes). In the first round of coding, participants from Belgium, China, and Italy mentioned, under the closeness metatheme, sub-metathemes such as “affection,” “cares about the student,” “cooperation,” “having fun together,” “open communication,” “respect,” “secure base,” and “trust” without any prompting by the reviewers or existing definitions of TSR. Similarly, in relation to the conflict metatheme, sub-metathemes like “dissatisfaction and negative feelings,” “doubting,” and “struggling relationship” emerged. Under the dependency, only one sub-metatheme, “encourage students’ independence,” was found. Furthermore, during this phase, specific countries mentioned distinct themes. For instance, “acceptance by teachers” was unique to Belgium and Italy, “gift-giving” was specific to China and Belgium, “greetings” only emerged in China, “interest in lessons” was only mentioned in China, “looking up to the teacher” was exclusive to Belgium and Italy, “regarding as a member of the family/friend” appeared solely in China, and “seeking practical help” was present only in Belgium and Italy.

After introducing the TSR concepts based on Pianta’s (2001) model, the second round of coding brought forth new ideas from participants of all three countries regarding the closeness metatheme. These new insights included concepts like “actively looks for contact,” “attunement,” “keeping track of the teacher,” “safe haven,” and “satisfactory relationship.” Additionally, the specific sub-metathemes identified in the first round, such as “acceptance by teachers,” “gift-giving,” “regarding as a member of the family/friend,” and “seeking practical help,” were also mentioned by participants from all countries at this stage. For the conflict metatheme, additional themes like “conflict resolution,” “fear,” “non-communication,” “non-conflict,” “non-cooperative behaviors,” and “unfairness” were identified. Interestingly, more sub-metathemes related to dependency emerged than in the first round, such as “asking for help constantly,” “confirmation seeking,” “demanding attention,” “emotional dependency,” “instrumental dependency,” “non-overreliance,” “physical closeness,” and “teacher improves students’ dependency.”

In addition to Pianta’s (2001) model, coders also identified “other dimensions” that pertained to social and emotional interactions between teachers and students. These dimensions yielded seven sub-metathemes, not perfectly aligning with the earlier categories. In the first round of analysis, themes like “authority,” “patience,” and “strictness” emerged across the three countries. Upon introducing Pianta’s (2001) model, new themes such as “distance” and “fairness” were mentioned. Although “increasing student motivation” was initially referenced by Chinese and Italian teachers, Belgian teachers also brought it up in the second round of analysis. Interestingly, the theme of “balance” was uniquely raised by Belgian teachers in both the first and second rounds of analysis.

Specifically, “authority” refers to teachers demanding student obedience, signifying an authoritative role. “Distance,” defined as the coolness or unfriendliness in the way that teacher/student behaves toward each other, was initially assumed to be a form of conflict or the opposite of closeness. However, certain site-specific themes related to this sub-metatheme could not align with the definition of conflict or closeness. Examples include “student shows no emotion or reaction when a teacher asks them about what happened or why a conflict happened,” “students are reluctant to invite teachers to participate in chats,” and “teacher needs to understand if students manifest affection naturally or if they want something in exchange” (for further information, please refer to Appendix 5). Instead, these themes may represent low levels of conflict and closeness, implying a more neutral emotional connection between the teacher and students. Similarly, “balance” (indicating the need to keep balance in the classroom), “fairness” (treating students impartially), “increasing student motivation,” “patience” (maintaining composure with students), and “strictness” (regarding many actions of students as unacceptable) were initially considered for inclusion in the closeness or conflict category. Nevertheless, after deliberation, all coders agreed to place these themes in the “other dimensions” category because balancing, fairness, increasing student motivation, patience, and strictness can exist without necessarily implying a close or conflictual relationship between a teacher and a student.

### 5.2. Cultural universality

#### 5.2.1. Teacher-student closeness

The analysis of cross-site themes revealed that teachers from Belgium, China, and Italy all made connections between several sub-metathemes regarding teacher-student closeness. These included the teacher’s perception that the student feels emotionally accepted by him/her and comfortable seeking practical help from him/her. Students may view the teacher as a secure base and safe haven, feeling a sense of safety in their relationship and seeking comfort and reassurance from the teacher. At the behavioral level, students may share personal experiences and opinions with the teacher, readily follow his/her suggestions, look up to him/her and view him/her as a role model, keep track of the teacher, or expect the teacher’s presence. Moreover, participating teachers articulated the positive emotional and social experiences shared by them and students. These experiences included verbal or physical expressions of liking (e.g., the student says “I love the teacher”; the teacher and student hug), satisfactory relationships (e.g., the student prefers his/her teacher to another teacher; the teacher feels emotionally involved in the relationship), as well as mutual respect and trust.

#### 5.2.2. Teacher-student conflict

Teachers across Belgium, China, and Italy perceived conflict in the relationship when students did not trust teachers, refused to communicate, or when teachers were seen as treating students unfairly or reprimanding them. Conflict was associated with negative emotions and social experiences, including dissatisfaction and negative feelings (e.g., explicit expressions of anger, implicit expressions such as sulking), and struggling relationships characterized by students’ disobedience and difficult interactions between teachers and students.

#### 5.2.3. Student’s dependency

When discussing dependency, teachers from Belgium, China, and Italy highlighted behaviors such as excessive proximity seeking, constant requests for help, attention-seeking, inappropriate physical closeness, and the need for constant confirmation. They also recognized instrumental dependency, where students extensively rely on teachers for help with schoolwork/tasks associated with their lessons. Teachers also emphasized the importance of fostering independence in students and providing them with the tools to solve problems independently, thus reducing their dependence.

#### 5.2.4. Other dimensions related to TSR

Belgian, Chinese, and Italian teachers identified several key TSR elements, including fairness, patience, increasing student motivation, and strictness. Participating teachers mentioned that fairness fosters trust and respect in the classroom, while patience creates a safe and supportive learning environment. Motivating students helps keep them engaged and focused, and strictness maintains discipline and reinforces classroom rules. According to the teachers, these elements can be distinct from teacher-student closeness or conflict, as a teacher can exhibit fairness or strictness without necessarily having a close or conflicted relationship with a student. For instance, Catho, a female Belgian teacher with 16 years of teaching experience, highlighted the balance between fairness and strictness, stating: “I am also quite strict actually, but I also try to be fair, so uh, yes, all right, I also hear that from my students who have been with me that they experience me as strict and fair.” Kaiwen, a male Chinese teacher with 8 years of teaching experience, emphasized the presentation of strictness without impacting TSR: “But they can feel when you love this group of students, although you keep scolding them, but they can also feel it will not feel you are very aggressive or feel you as if he does not like you, yes, I feel that is from the heart.” In a similar vein, Heidi, a female Italian teacher with 33 years of teaching experience, stressed the importance of communicating love even in criticism, noting: “Even in criticism they must see that you do it just for love, that you do it just for interest, that you don’t do it to judge or criticize.”

### 5.3. Cultural specificity

#### 5.3.1. Teacher-student closeness

Teachers’ responses to students’ emotional and behavioral cues, such as “attunement,” vary across countries. Belgian and Italian teachers demonstrated attentiveness to students’ emotions and explicitly responded to them by asking about their wellbeing, showing empathy, and actively listening. In contrast, Chinese teachers tend to react to students more implicitly, using strategies such as making eye contact with emotional expression and providing constructive reprimands.

In the way teachers perceive and demonstrate their care for students in the classroom, Belgian teachers showed care by being more attentive to students’ feelings (e.g., being a listening ear for pupils and giving them space to say things), whereas Chinese teachers show care by being more physical nurturing and instructive toward students (e.g., caring about students’ physical health; adjusting teaching methods according to their personality). Italian teachers place equal importance on both the emotional and physical wellbeing of their students (e.g., letting students understand he or she cares about them by means of physical contact or reaching out with a phone call when a student is feeling sick).

Chinese and Italian teachers mentioned that greetings can be seen as an indicator of closeness. Chinese teachers talked more about teacher-centered greetings (e.g., the student said “Good morning, teacher” and the teacher reacted to the student’s “Hello”). In contrast, Italian teachers mentioned student-centered greetings more often (e.g., the teacher welcomed students at the beginning of the day and said goodbye to the students one by one at the end of the year). Although Belgian coders agreed that greeting each other in schools is common, Belgian teachers, in the interviews, did not directly link greetings to closeness.

Giving gifts was seen as a significant means of showing respect and gratitude. However, the form of these gifts can vary across different cultures. For instance, Belgian and Italian students typically gave their teachers gifts like drawings and letters. In contrast, Chinese students not only presented their own drawings but also gave food, like snacks or fruits, to their teachers, who sometimes reciprocated by preparing food for the students.

Although Belgian and Italian teachers showed an active interest in students’ lives, noticing things like new backpacks or haircuts, Chinese teachers did not explicitly mention this aspect.

Regarding students’ interest in the lessons, Chinese and Italian teachers often connect it with fostering a positive TSR. For example, Gerlanda, a female Italian teacher with 19 years of teaching experience, mentioned, “When some child (said)… ‘Teacher, today I had fun when we did the lesson.’ then, of course, it makes me feel particularly in tune.” Similarly, Kaiwen, a male Chinese teacher with 8 years of teaching experience, indicated, “Intimacy, maybe he will like you so much in gym class that he will come to play with you in gym class.” However, even after receiving a prompt (i.e., the definition of closeness), Belgian teachers did not refer to this link.

Chinese and Italian students viewed their teachers as part of their extended family, often addressing them as “mother” or “grandma.” Belgian teachers referred to this aspect more indirectly; for example, Joke, a female Belgian teacher with 39 years of teaching experience, said: “I like to mother them too, they are still small. I always like to do that. If they have an ache or so, that you give them attention and, yes, they always come to you.”

#### 5.3.2. Teacher-student conflict

Although teachers from different countries perceived non-cooperative behaviors such as not following instructions and disrupting class as teacher-student conflict, only Chinese teachers considered students arguing with teachers as a part of the conflict. For example, Yuxue, a Chinese female teacher with 5 years of teaching experience, said: “Conflict is mainly manifested in, for example, the child made a mistake, but when this mistake involves people other than him, if the teacher just criticizes him, he will argue with the teacher, most of his arguments, is to blame others, or to divert the teacher’s attention.”

Furthermore, Belgian teachers did not mention students’ fear of teachers, and a few Italian teachers viewed it as a negative or unwanted aspect of the TSR. On the contrary, Chinese teachers may perceive the concept of “fear” in the classroom environment differently, considering it as a neutral or even desirable aspect. For example, Shiliu, a female Chinese teacher with 27 years of teaching experience, indicated, “I used to have a colleague who was harsh, once he was in class, he hadn’t arrived in the classroom, the students were sitting and waiting for him, waiting for his class, and his students were very active, but I don’t know what method he used to make the atmosphere very good when the students should be in class, but once the class was over, the students were especially afraid of him.”

Regarding (corporal) punishment, Chinese and Italian teachers may resort to punitive measures, such as physical discipline with a ruler or exclusion from activities, in response to students not following instructions, which can contribute to teacher-student conflict. In comparison, there was no mention of this approach by Belgian teachers.

In terms of the frequency of conflict, Chinese and Italian teachers were more likely to perceive no conflict compared to Belgian teachers. For example, Anhe, a female Chinese teacher with 8 years of teaching experience, stated, “I don’t think I’ve seen any long-term conflicts between teachers and small children. Because children are still very simple, …, in my years as a teacher, I basically did not encounter conflict between teachers and children, like the conflict between adults, anyway, I have not encountered.” And Ace, a female Italian teacher with 4 years of teaching experience, said, “No, no, I have never had conflicts, no, let’s say that the conflicts usually… maybe because up to the primary school, you know, hardly with children so young you can have a conflict, maybe already in the middle school, in the high school, but not with the really young children.”

Conflict resolution approaches also exhibited variations, with Belgian and Italian teachers employing various methods involving direct communication, open discussion, and collaborative problem-solving. For instance, Femke, a female Belgian teacher with 8 years of teaching experience, expressed her approach to conflict resolution: “I find quarrels very, very difficult because they always happen behind our backs, …, but I try to listen to both parties and try to mediate a little of that, …, also ask them how are we going to do that and let them express this to each other after the event, that they themselves try to talk it out in a decent way.” Similarly, Grace, a female Italian teacher with 5 years of teaching experience, shared her strategy of communicating and building trust with a child with problematic behaviors after conflict: “Yes, I had a child who has big difficulties, big family problems and so he tends to externalize behaviors. (I tried) to calm him down, I take him near me, maybe it happened many times to speak to him and to say ‘But listen, but does it seem a behavior (…).”’ “No, you are right” he says to me, and I remember that I said to him “Do you trust what I tell you. that is, when I speak to you, do you trust what the teacher says, don’t you trust your teacher?” and he “Yes, yes” “here, then let’s avoid, maybe, you will see that it is not an attitude that will help you this.” However, Chinese teachers did not explicitly touch upon these conflict resolution approaches in their interviews. Although they did discuss how they communicated with students when they made mistakes, the teachers associated this more with teaching than with resolving conflicts with the students.

#### 5.3.3. Student’s dependency

Chinese and Italian teachers showed overlapping themes between closeness and dependency, where certain aspects could be interpreted as either teacher-student closeness or dependency. In response to both Q2 (regarding behaviors representing closeness) and Q4 (regarding behaviors indicating dependency), Chinese teachers identified actions such as “student sees the teacher as a parent” that fell within the scope of both concepts. For instance, when discussing Q2, Yuxue, a female Chinese teacher with 5 years of teaching experience, remarked “He (the child) will suddenly call me mom, yes, and then just like that, it is a kind of intimate embodiment.” Similarly, Kaiwen, a male Chinese teacher with 8 years of teaching experience, described it in response to Q4 “Yes, especially for dependent children, I feel that for me this is more obvious because I have more contact with him, sometimes (for the child, I am) more like his father, on the one hand, I am a teacher… He is the lack of love.” Similar parallels emerged in the Italian participants. The theme that “students want to be psychically close to the teacher during break time” could be construed as either closeness or dependency, depending on the teacher’s perspective. Ace, a female Italian teacher with 4 years of teaching experience, explained in response to Q2 “then it happens usually during the break time, because they eat at school and do the break time, …, so the moment in which they really are closer is during the break time, they see me there, they play, they approach.” Meanwhile, Paula, a female Italian teacher with 14 years of teaching experience, addressed Q4 “Well, there was a child, or rather a couple of children, who were particularly dependent on the teachers. One little girl, for example, during the whole recess period wouldn’t let go of me, despite my interventions to try to make her play and in spite of the children’s interventions asking for her presence.” Certain Italian teachers even acknowledged unintentionally reinforcing students’ dependency. Aisha, a female Italian teacher with 15 years of teaching experience, shared “Just a minute, I have to do everything, just a minute’ I’ll check on all of you.” This hinted at catering to children’s need for dependency. It is worth noting that while these intriguing overlaps were observed among Chinese and Italian teachers, Belgian teachers did not exhibit a similar tendency.

Even more intriguingly, some Chinese and Italian teachers noted that, in the years of their teaching, they did not come across any students who exhibited over-reliance tendencies. Taizhe, a male Chinese teacher with 17 years of teaching experience, raised doubts about the concept of dependency as provided and mentioned that he had not encountered any dependent students. He expressed “I don’t really understand what dependency means, because that’s your definition, because for me I don’t have this student, I never feel that the student has all kinds of dependence. If the student is willing to follow me, he must have his own will and needs.” Similarly, Grace, a female Italian teacher with 5 years of teaching experience, conveyed “So I don’t have anyone who’s really that morbidly attached, I don’t even find it to be really.” Notably, Belgian teachers did not offer similar comments, suggesting an absence of dependency in their interviews.

#### 5.3.4. Other dimensions related to TSR

Teachers from all three countries highlighted the importance of being authoritative figures, but differences were observed, particularly among Belgian teachers. Belgian teachers emphasized the need to maintain a balance, combining authority with playfulness and warmth. This sentiment was echoed by Sara, a female Belgian teacher with 35 years of teaching experience, who indicated “I don’t want to be too authoritarian. I want them to feel comfortable, to be able to say what they want, to have the courage to speak and the courage to ask for something, too. So that you have to be nice to them and not too strict.”

Numerous Belgian teachers highlighted the importance of balancing various elements, such as being a confidant while maintaining appropriate distance and managing work-play balance. For example, Michiel, a male Belgian teacher with 6 years of teaching experience, told the interviewer, “As a male teacher, sometimes it’s hard to be a bit (hard to draw certain boundaries) toward those young children. They do give hugs and they do tell you a lot where you want to also, yes, no, not have a wrong image, or you still want to keep a certain distance because those still come and hug a lot and, and hang on to you and that’s a difficult one to draw certain boundary.” Relatedly, Lieze, a female Belgian with 6 years of teaching experience, was trying to balance authority and being a warm teacher: “Still, not something I immediately come up with. It’s very important that they feel good with you. I think that’s the most important thing. That they know what’s allowed and what’s not. And that is also a difficult one for me because I am often not strict enough.” However, these considerations were less prominent among Chinese and Italian teachers.

The concept of distance between teachers and students, reflecting coolness or unfriendliness in interactions, emerged in interviews with Belgian, Chinese, and Italian teachers. Some aspects of distance were universal, such as lacking emotional connection. For instance, Belgian teachers noted that students showed no emotional response when questioned about conflicts, Chinese teachers observed students’ reluctance to open up during communication, and Italian teachers mentioned a preference for students not discussing private matters. However, Chinese teachers also associated distance with student behavior related to a lack of greetings or interest in lessons. For example, Meixi, a female Chinese student with 4 years of teaching experience, found that “Third grade and lower grade students may say ‘I don’t like you as a teacher, and then I will not like the class you teach, and then when I am in your class, I will do my own thing, or I will not listen to the class.”’ Italian teachers mentioned feeling distant when uncertain about students’ genuine affection, as it did in the case of Ace, a female Italian teacher with 4 years of teaching experience, who said, “I have to understand where the limit is between a gesture made with affection and a gesture made because maybe they want something in exchange from us teachers.” However, these connections were not made among Belgian teachers.

## 6. Discussion

This study investigated the universality and specificity of how TSR is understood and practiced by teachers in Belgium, China, and Italy. Among the three countries, there was a consistent alignment with Pianta’s (2001) model in terms of their conceptualization of closeness and conflict, albeit with certain nuances in how these concepts were conveyed across different countries. The concept of dependency, however, exhibited cultural differences in interpretation, consistent with quantitative findings from previous studies that suggest the association of dependency with closeness can be either negative or positive, depending on the cultural context of individualism or collectivism (Webb and Neuharth-Pritchett, 2011; Tsigilis et al., 2018; Gregoriadis et al., 2021; Vahidi et al., 2022). Furthermore, the study identified additional dimensions beyond Pianta’s (2001) model, such as authority, balance, distance, fairness, increasing student motivation, patience, and strictness. Among these dimensions, fairness, increasing student motivation, patience, and strictness exhibited similar conceptualization and manifestation across cultures, whereas authority, balance, and distance showed variations.

### 6.1. Cultural universality of closeness, conflict, and dependency

In general, this study provided evidence supporting the universality of the concepts of closeness and conflict across cultures. This evidence was also derived from the analysis of the first-round coding, specifically the responses to the free recall questions. This finding aligns with previous findings that the STRS, particularly the dimension of closeness and conflict, has consistently shown reliable validity in measuring closeness and conflict (e.g., Koomen et al., 2012; Milatz et al., 2014). This supports the idea that TSR may reflect the attachment behavioral system, indicating that teachers can serve as *ad hoc* attachment figures and fulfill some of the functions of an attachment relationship across cultures (Verschueren and Koomen, 2012). Attachment theory identifies secure and insecure attachment types, such as anxious-ambivalent and avoidant attachments (Ainsworth et al., 1974). The current findings indicate that the patterns related to teacher-student closeness, such as the extended secure attachment, can be observed across cultures. This is indicated by students feeling safe with teachers, seeking comfort from them in times of trouble, actively looking for contact and keeping track of them, and aligning with the attachment behavioral system components, including safe haven, secure base, proximity maintenance, and separation distress (Bergin and Bergin, 2009; Posada et al., 2013). In contrast, teacher-student conflict and student dependency indicate insecure attachment between the student and the teacher, including a lack of trust, negative feelings, and excessive proximity seeking (Bergin and Bergin, 2009; Posada et al., 2013).

### 6.2. Cultural specificity of closeness, conflict, and dependency

Despite a similar understanding of closeness and conflict across cultures, there were variations in their expressions. This further supports the idea that partial strong measurement invariance was found using STRS between cultures, indicating that certain items may have different meanings across cultures (Chen et al., 2019). Moreover, there were inconsistencies in the interpretation of dependency across cultures. There are two factors that may explain the differences observed between cultures.

#### 6.2.1. Culturally normative practice

TSR practices can be interpreted differently depending on cultural normativeness (i.e., the degree of acceptance of behavior within a particular culture; Lansford, 2021). Belgian teachers, in comparison to Chinese teachers who tended to emphasize instrumental support and attending to educational needs, more frequently displayed emotional care as a form of warmth. The findings align with prior cross-cultural research on parent-child warmth, highlighting variations in expressions of warmth in different cultural contexts. Chinese immigrant mothers emphasized nurturance and instrumental support, while European American mothers demonstrated warmth through direct physical and verbal expressions (Cheah et al., 2015). Interestingly, Italian teachers exhibited both forms of warmth. This finding further supports the claim that differences in the expression of warmth may be attributed to the contrasting values of collectivism and individualism, particularly considering the mixed characteristics of individualistic and collectivistic values in Italy.

A similar pattern emerged in the sub-metathemes of greetings and gift-giving as a form of social communication, with Chinese and Italian teachers placing more emphasis on closeness, whereas Belgian teachers do not demonstrate the same emphasis.

Regarding conflict, among Chinese and Italian teachers, physical punishment may be used as a disciplinary measure, while in Belgium, it is considered inappropriate (Global Initiative to End All Corporal Punishment of Children, 2021). In addition, it was found that the fear of the teacher, which can be interpreted as respect for teachers, may be seen as necessary and even desirable among Chinese teachers. However, in Belgium and Italy, it is considered essential to maintain authority while simultaneously fostering positive and supportive relationships with students, with less emphasis on fear. Taken together, these results suggest that the variations of expression of TSR were observed between cultures. As Lansford et al. (2018) found that parenting practices could result in more favorable child outcomes when they conform to cultural norms, the present study’s findings may illuminate the significance of normative teaching practices, which could prove beneficial for future classroom interventions across different cultures.

#### 6.2.2. Collectivistic versus individualistic value

Although greetings hold significance in both China and Italy, differences in greetings emerged between Chinese and Italian teachers. Chinese teachers highlighted teacher-centered greetings, whereas Italian teachers referenced student-centered greetings. This disparity might reflect distinct cultural interpretations of sensitive responsiveness in caregiving or teaching (Keller et al., 2018; Mesman, 2018). Initially defined by Ainsworth et al. (1974), sensitive responsiveness refers to a caregiver’s ability to notice, interpret, and respond promptly and appropriately to an individual child’s signals. This concept has been extended to TSR, where teacher sensitivity also predicts the quality of the relationship (Spilt and Koomen, 2022). However, it has been observed that caregiver or teacher sensitivity may not be present in some cultural contexts, as it does not align with local values (Keller et al., 2018). In individualistic cultures, caregivers or teachers are expected to adopt the child’s perspective, and interactions are primarily dyadic, while in collectivistic cultures, caregivers or teachers often guide children to consider the perspectives of others, fostering their ability to consider others’ needs and desires (Keller et al., 2018).

The aspects of non-conflict and conflict resolution, part of teachers’ perceptions of conflict, were found to be culturally distinct. Collectivistic cultures, such as China, tend to avoid conflict and highly value harmony, in which people may be more likely to avoid expressing disagreement or criticism openly and instead use indirect communication and non-verbal cues to convey their conflictual thoughts and feelings (Han, 2017). So, the conflicts might be more difficult to observe and consequently solved. By contrast, in individualistic cultures, such as Belgium, direct communication and the expression of dissenting opinions could be more common and even encouraged (Hofstede et al., 2010). This can lead to a greater likelihood of conflict but can also lead to more open communication and diverse conflict resolutions. Particularly, teachers from Italy referred to both non-conflict and conflict resolution, which further emphasizes the influence of both collectivistic and individualistic values. This evidence, again, holds particular significance, given the coexistence of these cultural dimensions within Italy (Hofstede et al., 2010).

Moreover, the findings of this study suggest that the values of collectivism versus individualism may play a role in explaining the cultural variations in the perception of concepts such as dependency among teachers. Although the current findings show that the teachers from the three countries mentioned similar expressions of student dependency, such as students asking for help constantly, confirmation seeking, demanding attention, and overly demanding physical closeness, it is worth noticing that the concepts of emotional dependency might differ across cultures. These differences may reflect divergent views of the self in relation to others in collectivistic and individualistic cultures. In collectivistic cultures, children may be taught that social relationships are important to the self, leading to a greater emphasis on interdependence (Kitayama and Salvador, 2017). For instance, as early as infancy, Japanese mothers tend to direct their children toward themselves, promoting interdependence in the mother-infant relationship. In contrast, United States mothers tend to direct their children toward objects in the environment, promoting independent exploration (Bornstein et al., 2012). Consequently, teachers in collectivistic cultures may encourage children to rely on adults for guidance and support, resulting in a greater tolerance for emotional dependency, and emotional dependency may be viewed as similar to closeness (Gregoriadis et al., 2021; Spilt and Koomen, 2022; Vahidi et al., 2022). In contrast, individualistic cultures prioritize independence and separation of the self from the social context. Therefore, dependency may conflict with the emphasis on autonomy and independent exploration, leading to a more apparent separation between closeness and dependency (Webb and Neuharth-Pritchett, 2011). These cultural differences may influence how emotional dependency is viewed as either adaptive or maladaptive.

### 6.3. Cultural universality of other relevant relational aspects

This study confirms Pianta’s (2001) model while expanding upon it by uncovering additional dimensions in TSR, highlighting that effective TSR involves a delicate balance of multiple factors of TSR that impact student learning, engagement, and wellbeing. Across cultures, a supportive classroom learning environment requires teachers to use effective teaching strategies such as fairness, increasing student motivation, patience, and strictness to organize classroom activities. While attachment theory describes TSR as an emotionally involved relationship, other theories, like interpersonal theory, provide additional frameworks for understanding it (Spilt et al., 2022). For example, Wubbels and Brekelmans (2005) proposed the model of interpersonal teacher behavior, which introduced the dimensions of dominance versus submission and opposition versus cooperation. In this model, teacher fairness can be understood as a form of teacher leadership characterized by high dominance and low to medium cooperation. Similarly, increasing student motivation and patience can be seen as a combination of high cooperation and low to medium dominance, while strictness may relate to high dominance and low to medium opposition (Wubbels and Brekelmans, 2005). These dimensions of teacher behavior have implications for the TSR and may influence how students perceive and respond to their teachers; for example, the promotion of child development through positive emotions and emotional security can be facilitated by reciprocity within TSR (Locke and Sadler, 2007).

### 6.4. Cultural specificity of other relevant relational aspects

Teachers from Belgium, China, and Italy demonstrated a non-emotional connection, referred to as “distance” between students and teachers. This concept may reflect avoidant attachment, characterized by a preference to avoid emotional closeness and downplay the significance of relationships (Ainsworth, 1979). Howes and Ritchie (1999), in a study involving 3,062 primarily underprivileged children, explored attachment to early childhood teachers and found similarities between avoidant attachment to teachers and parent-child relationships. O’Connor and McCartney (2007) also indicated that students exhibiting an avoidant attachment style might feel uncomfortable with excessive emotional closeness with their teachers, favoring a more distant stance. In this context, avoidant children did not engage with teachers in open conversation, often eluding their notice; in times of distress or agitation, they refrained from seeking the teacher’s assistance (Bergin and Bergin, 2009). The same pattern was observed in the identification of distance in the present study, suggesting a potential TSR concept for further exploration. Moreover, the expression of distance varies among teachers from Belgium, China, and Italy. Although teachers from these countries all perceived limited relationships and reduced open communication, Chinese teachers also emphasized greetings and course enjoyment of students, while Italian teachers expressed concerns about potential manipulation by students. This, again, supports the idea that the interpretation of TSR practices can vary based on cultural norms (Lansford, 2021).

Although teachers from Belgium, China, and Italy emphasized authority, Belgian and Italian teachers emphasized warmth more. Particularly, Belgian teachers strove to balance their teaching strategies to achieve this goal, suggesting they may focus more on equality and cooperation between the teacher and student than Chinese and Italian teachers. These differences can be attributed to variations in beliefs about teacher authority among individuals from different cultural backgrounds (Lai et al., 2015). In Eastern cultures, such as China, where Confucian respect emphasizes structure, order, and hierarchical deference, teachers are regarded as experts in their subject areas and as role models and moral agents. Consequently, their actions are seldom questioned by students (Gao, 2010). On the other hand, Western cultures, such as Belgium, emphasize individualism and democratic decision-making, which can lead to a more egalitarian relationship between teachers and students. Students in these cultures are encouraged to express their opinions and respectfully challenge authority figures, including teachers (Hall Haley and Ferro, 2011).

### 6.5. Theoretical, methodological, and educational implications

This study contributes to the theoretical understanding of attachment theory and underscores the need for culturally sensitive measurements by demonstrating its relevance and adaptability in diverse cultural contexts. On the one hand, enhancing the scale could involve incorporating both culturally universal practices in the STRS (Pianta, 2001), such as “the child feels understood by the teacher” and “the child had fun with the teacher,” as well as culturally specific items like “the child often greets the teacher.” On the other hand, when assessing some culturally sensitive concepts, such as dependency, careful consideration should be given to the cultural nuances and mixed meanings associated with this dimension. Future studies should employ culturally appropriate measures to capture teachers’ and students’ unique cultural perspectives and experiences of teachers and students, which is crucial for comprehensive cross-cultural investigations.

Recent research underscores disparities in psychological intervention engagement and outcomes among culturally diverse groups, emphasizing the need to incorporate cultural factors into conceptualization and implementation (Sanchez et al., 2022; Huey et al., 2023). Tailoring TSR practices and interventions to align with cultural norms is essential for achieving optimal results, particularly as the cultural fit between students and teachers can positively influence academic performance and behavioral outcomes in school (for review, see Redding, 2019). The findings of the current study contribute to a deeper understanding of TSR and facilitate culturally appropriate classroom interaction. For instance, in a classroom with a collectivistic student and an individualistic teacher, the teacher might discern that the child’s dependency could, from the child’s standpoint, indicate a sense of proximity or closeness with the teacher. Based on this, adjusting the teacher’s expectations and gradually encouraging the child’s independence can be part of fostering positive TSR. The findings of this study could promote culturally sensitive mental representations among teachers and facilitate positive TSR across diverse backgrounds within and across countries.

### 6.6. Limitations and future studies

The in-depth interviews of this study have enhanced the understanding of participants’ perspectives and experiences (Maier et al., 2004). Moreover, the metatheme analysis has provided valuable insights into the complexities of TSR across cultural sites (Wutich et al., 2021; Trainer et al., 2022). However, it is crucial to acknowledge the diversity of cultural values within the countries studied. In future data collection, including specific information related to individualism and collectivism would be valuable. Döge and Keller (2014) found a low correlation of (cultural) socialization goals between migrant mothers and teachers, suggesting cross-cultural disparities within the country. By encompassing the distinct (cultural) socialization goals of both teachers and parents, researchers can gain insights into how culture shapes the beliefs and expectations of these stakeholders. This understanding may become particularly crucial when there is a mismatch in cultural backgrounds between the teachers and students (Redding, 2019). For instance, consider how a Belgian teacher instructs a class primarily composed of children from a more collectivistic culture, and how this cultural difference might impact TSR. Additionally, it is worth noting that the data collection in this study was limited to teachers’ perspectives on TSR. Previous studies have produced inconsistent results concerning the agreement between teachers and children’s perceptions of TSR (Hughes and Cavell, 1999; Hughes, 2011; Jellesma et al., 2015; Koomen and Jellesma, 2015), suggesting that mental representations might differ between these perspectives. To authentically capture the feelings and interpretations of TSR, including the voices of children in future research, is essential. Lastly, given the qualitative nature of the study, it was not feasible to conduct further analysis to investigate the potential influence of the characteristics of school and teachers, such as school system, classroom size, gender, teaching experience, and teacher sensitivity, on their perception of TSR. Subsequent research on the cross-cultural applicability of TSR measures could benefit from empirical data on individualism and collectivism, as well as examining the impact of school’s and participants’ characteristics on TSR perceptions.

## 7. Conclusion

This study aimed to explore the cultural universality and specificity of TSR concept and manifestation by conducting semi-structured interviews with teachers from Belgium, China, and Italy, representing varying degrees of individualism and collectivism. The findings illuminate how TSR is perceived and expressed across these different cultural contexts. Understanding these cultural differences and similarities in TSR is crucial for developing teaching practices that are sensitive to diverse cultural backgrounds. This study lays the foundation for future research to build upon, providing a basis for more comprehensive insights and effective strategies to promote positive TSR in diverse cultural contexts.

## Data availability statement

The datasets presented in this article are not readily available because the Social and Societal Ethics Committee of KU Leuven has advised against sharing the dataset due to the possibility of identifiable aspects in the transcripts. Requests to access the datasets should be directed to canmei.xu@kuleuven.be.

## Ethics statement

The studies involving humans were approved by the KU Leuven Social and Societal Ethics Committee. The studies were conducted in accordance with the local legislation and institutional requirements. The participants provided their written informed consent to participate in this study.

## Author contributions

CX: Conceptualization, Data curation, Formal analysis, Methodology, Writing – original draft, Writing – review and editing. MH: Conceptualization, Methodology, Supervision, Writing – review and editing. GD: Formal analysis, Writing – review and editing. SP: Formal analysis, Writing – review and editing. RL: Writing – review and editing, Formal analysis. SS: Writing – review and editing. DR: Conceptualization, Methodology, Writing – review and editing. DB: Conceptualization, Methodology, Project administration, Supervision, Writing – review and editing.
