# Supplementary material for: Cultural universality and specificity of teacher-student relationship: a qualitative study in Belgian, Chinese, and Italian primary school teachers
Source: Front Psychol. 2023 Nov 14;14:1287511. doi: 10.3389/fpsyg.2023.1287511 (PMC10682107; doi:10.3389/fpsyg.2023.1287511)
Supplement: Supplementary file 1 [file Data_Sheet_1.docx]

**Appendix 1 Cultural reflection**

Coder 1: I believe my cultural background as a female psychology student from a western country certainly influences the research process, interviews, and analyses. As I have interviewed other western teachers, it gives the benefit of more easily understanding what they mean and certain definitions they use. However, the downside is that this may cause me to ask less in-depth questions to further determine the meaning of the definitions they use, as I may believe I already understand what they are trying to say. As someone from a western, more individualistic culture I also have more western views on certain aspects of the teacher student relationship, such as closeness, conflict, and dependency, which can also influence the way I conducted and understood the interviews and the codes I choose in the thematical analysis. Furthermore, I do believe my background as an international student also has an impact, as perhaps my exposure to different cultures, and teachers of different cultures may have given me a firsthand experience on the teacher student relationship and how this may differ between students and teachers of different cultures.  Finally, I believe my background as a psychology student also colors my analyses. Due to the subjects I have taken and the research I have done and articles I have read in writing my thesis it may be harder not to let myself be influenced by existing theoretical perspectives (such as attachment theory, self-determination theory, etc).

Coder 2: I think that your cultural background can influence the research process in an accidental bias in which theories you include or see as important in your study. Usually, I see theories constructed by European or American researchers. While there must be other theories out there. Also, during the interview, a participant can ask for an example, and the example that I give can influence their answer. And my reaction to certain answers can also influence their next answers. There are certain thing in my culture that we see as normal in a teacher-student relationship, but this can be different in a different culture, so my reaction is based in my cultural background.

Coder 3: My cultural background may affect the coding process. First, as a researcher from Chinese culture, I am more familiar with teacher-student relationships in the context of collectivism. I may not perceive some narratives as codable items which are salient in other cultures. I probably do not realize some common notions in China need further interpretations for people from other backgrounds. Second, the concept of teacher-student relationships is changing over time. I may unconsciously use my previous school experiences (around 20 years ago) to look into current situations. Third, my previous theoretical knowledge regarding teacher-student relationships also shaped my insight into the interviews. It is challenging to leave those typologies I know aside and take a new perspective.

Coder 4: As a psychologist trained in quantitative research methods, and a researcher from a Chinese cultural background with knowledge of cross-cultural investigations, my approach to interviewing and coding may be influenced in several ways. Firstly, due to my training in quantitative methods, I may tend to prioritize representation over deep interpretation when conducting qualitative analysis. Secondly, given the cultural expectation in China that teachers prioritize teaching over emotional caretaking, I may place more emphasis on social interactions than emotional interactions when observing teacher-student interactions. It is also possible that I may unintentionally overlook themes that are common in Chinese culture but may be of interest to researchers from other cultural backgrounds. Thirdly, my understanding of cultural differences in parenting, particularly the impact of collectivist and individualistic values on parent-child interactions, may offer valuable insights when comparing teacher-student relationships across cultures. However, I acknowledge the possibility of inadvertently neglecting other important aspects of teacher-student interactions.

Coder 5: I think my cultural background influence me in all the research process firstly because, during the interview, I explored deeper the most visible and common aspects and contents teachers presented (so, for example, typical teacher-child issues and problems that everyone knows in Italian context), and, especially, I explored more aspects and contents presented by more than one teacher or by the most teachers. I think this cultural bias will influence me during the analytical and coding decision because maybe I'll consider more important the most common and expected and, at the opposite, the most uncommon and unexpected behaviors and aspects of general Italian relations between teachers and children, risking to ignore the average aspects (aspects and behaviors that in my cultural view could be not relevant but maybe for other cultural views they could be). About the examples, one thing I realized the most teachers are focused on is the need to be empathic with the children or between them, and so, understand the needs of every single child and consideration of their individuality and individual exigencies, and the importance of behave in a specific way basing on their differences. Other typical and common elements are that teachers commonly listen to children about their any type problem and try to help them giving advices, that children hug teachers, teachers always want to involve children in interesting activities, and finally they emphasize a lot their strong emotional involvement in the relation.

Coder 6: As a female educational sciences student I believe my cultural background will influence the decisions I make in every part of the research process. I have grown up with the western views on illness/differentness. Illness is in the western society less seen as ‘depending on context’. Another example could be the egocentrism of the ‘self’. Westerners are described as autonomous and independent. It is a much more individualistic culture. During my internship I will need to be cautious as to not only view certain data in the perspective on my own culture, but look at it from multiple perspectives and continuously reflect on the assumptions I make. Growing up, I mostly came into contact with white populations. There are certain exceptions, which I will not discuss since there are few. I went to a mostly white school and even at university, studying educational sciences, I didn’t come into contact much with other cultures. The majority of students studying educational sciences are white, female, and have high SES. Nevertheless, the view I now have on different cultures, has also been shaped by that very same education. In high school I studied human sciences which was divided into behavioral sciences (“gedragswetenschappen”) and cultural sciences (“cultuurwetenschappen”). The latter also discussed other cultures and addressed situational aspects of minority-groups, though a bigger amount went to politics, communication, ethics, art, etc… which was all focused on my own culture. It then was a logical next step to study educational sciences at university to broaden and deepen my knowledge on people. Part of that education has been on intercultural education as well. In other words, I do have a background on how people can be different and have different ideas about the world. It also needs to be said that educational sciences focuses more on the context people live in, in comparison with psychology. In psychology the focus lies on the individual. That nuance also shapes the view I have on people. Another example of that can be found at my last internship. I was an intern at a student counseling center and we came into contact with minority-groups a lot. Some parents of children didn’t understand Dutch so we often hired a translator. Languages that I have come across are mostly Turkish and Dari. A prominent value at a student counseling center is strengthening minority-groups. We try to put in more of an effort since research suggest they can be at risk of falling behind. Furthermore, we are deciding too fast to transfer them to special education. This is something I have also been reflecting on during my last internship since it is very important that we give them all the chances they can get to lessen the gap with other pupils before we send them off to special education. The background I just described can influence for example data collection. Since Belgium is a multicultural country it is important to reflect on the methods for data collection. People from minority-groups in Belgium appreciate closeness. They like to reflect on socio-cultural issues in groups of people with the same background. It is sometimes hard to involve them each individually because they are less trusting of Belgian professional aid workers. This underlies the importance of working with aid workers of another culture. The same can also be said for doing research. A multicultural research group can be advantageous for both respondents as researchers. I’ve elaborately discussed this in my previous reflection as well as the concept of translation.

**Appendix 2 Cross-site Metatheme: Conceptualization and Manifestation of Closeness**

| Sub-metatheme  (Definition of sub-metatheme) | Sites-specific Themes (1st round) | | | Sites-specific Themes (2nd round) | | |
| --- | --- | --- | --- | --- | --- | --- |
|  | Belgium | China | Italy | Belgium | China | Italy |
| Acceptance by teachers  (students feeling understood by the teacher and the possible actions that can cause this) | The student should feel accepted by the teacher. | (Not mentioned) | The student feels understood by the teacher. | The student knows that they can share their emotions in class or with the teacher (e.g. that they will not be laughed at, that no one will get angry). | The student is happy to be understood and affirmed by the teacher.  Teacher accepts what student teaches to them. | The student feels accepted and free with the teacher.  The student feels listened to and understood by the teacher. |
| Actively looks for contact  (The teacher/student initiates communication) | (Not mentioned) | (Not mentioned) | (Not mentioned) | (Not mentioned) | The student looks forward to meeting the teacher.  The student is willing to spend time with the teacher.  The student invites teacher to his home. | The student wants to show the teacher what he/she did.  The student comes next to the teacher in order to talk with him or her.  The student talks to teacher outside the classroom. |
| Affection  (Student/teacher verbally or physically express that he/she likes the teacher/student) | The student shows that they like the teacher. | The student loves the teacher.  Teacher loves their students.  Teacher and student hug. | Teacher smiles to students.  Teacher loves students.  Teacher makes students feel loved.  Teacher likes some students.  Teacher prefers to communicate and enter into a relationship with students he or she likes.  Affection manifestation.  Hugs.  Physical contact. | The student shows affection to a teacher (e.g. a tap on the shoulder, a hug,..).  The student shows that he/she likes the teacher (e.g. by telling things). | The student says “love the teacher”.  The student initiates physical contact with the teacher (e.g., hug).  The student is willing to have physical contact with the teacher. | The student asks for physical contact (e.g., hug each other, the student seats on the teacher's lap).  The student holds the teacher's hand.  Teacher has physical contact with the student (e.g., the teacher caresses student's face).  Teacher and student are happy when they hug among each other.  Teacher feels closer to students turning around the classroom.  Teacher gives a pat on the shoulder to the student.  Teacher hugs students.  Teacher strokes students’ hair.  Teacher provides physical contact.  Teacher puts students next to him or her. |
| Attunement  (The teacher is in tune with what the student is feeling) | (Not mentioned) | (Not mentioned) | (Not mentioned) | Teacher being attentive to the student’s emotions and responding to them (e.g. by asking how things are going). | Teacher makes emotional eye contact with the student.  Teacher considers the psychological needs of the student and reproach them in a positive way. | The student perceives teacher's closeness and empathy.  Teacher feels students’ pain.  Teacher is empathic towards students.  Teacher always perceives synergy with students. |
| Cares about the student  (Teacher taking into account the emotions, physical health and wellbeing of students) | Being attentive to the students’ feelings and mood.  Being an empathetic teacher. | Teacher expresses understanding of student misbehavior.  Teachers educate students according to their personality.  Teacher and student are happy together.  Teacher is happy to accept the influence of students on him or her.  Teachers put themselves in the shoes of their students to understand their behavior.  Teachers take the initiative to break the ice.  Teachers treat students gently. | The student and teacher understand what they require from each other.  The student is cheerful with the teacher.  The student feels positive emotion towards the teacher.  The student feels the teacher is happy.  The student feels the teacher is passionate about their work.  The student understands what the teacher feels or wants without speaking to each other.  Empathy between teacher and student.  If students do something wrong, teachers let them understand it's a bad thing, but they're not bad people.  Teacher asks students how or what they are feeling.  Teacher bases his or her relationship with students on their emotions and experiences.  Teacher cares about students’ emotions.  Teacher cares about emotions.  Teacher feels students are happy.  Teacher has an empathetic attitude.  Teacher is emotionally involved in the relationship.  Teacher is happy and satisfied with their students’ emotional competence.  Teacher is sad to leave students at the end of the school year.  Teacher pays attention to students’ mood.  Teacher lets students understand their emotions and those of others.  Teacher takes care of students.  Teacher tries to understand students’ emotions.  Teacher understands students’ frailties.  Teacher has a feeling of tenderness towards students. | Teacher fosters the student.  Being a listening ear for pupils and giving them space to say things (e.g. by providing an individual moment, by asking if something is,..).  Creating opportunities for the student to show how it feels (e.g. working with notes read by the teacher, working with colors to show how the student feels,..).  Showing students that you are there for them (e.g. being physically close, asking how they are doing). | Teacher cares about students' physical health.  Teacher takes care of students' living.  Teacher considers the psychological needs of students and reproaches them in a positive way.  Teacher approaches the student. | Teacher brings students outside during the breaktime.  Teacher call students when they're feeling sick.  Teacher cares about students.  Teacher lets the students understand he or she cares about them by means of physical contact.  Teacher prepares a hot tea for a student presenting with a stomach ache. |
| Cooperation  (Teacher and student working together smoothly, or student follows teacher's suggestions readily) | Adapting lesson structure (e.g. working in small groups, individual work, playful work, etc).  The student works hard in class which makes the teacher enjoys teaching. | The student is willing to work with teachers.  The student actively participates in classroom activities.  The student accepts what the teacher is teaching.  The student interested in courses and the teachers. | The student cooperates with the teacher.  Teacher lets students collaborate among each other’s.  The student doesn't go against the teacher and attend easily his or her activities.  Teacher acts in a way to involve all students. | Giving the student the room and time to self-regulate. | The student actively answers teacher questions in class.  The student is willing to listen to teacher criticism (reasoning).  The student is willing to help the teacher.  The student is happy to respond to teacher words. | The student listens to the teacher.  Teacher explains to students the reasons of his or her acts.  Teacher replies to students' storytelling. |
| Gifts giving  (Teacher and student give gifts to each other) | (Not mentioned) | Gifts for teachers from students | The student makes drawings for the teacher.  The student writes letters to the teacher expressing their love to the teacher. | The student shows that he/she like the teacher (e.g. by making drawings,..) | The student shares food with teachers.  The student gives small gifts to teachers.  Teacher shares food with student.  The student volunteers to dance for teacher.  Teacher makes food for students. | The student gives teacher a paper as gift in which they wrote something to thank them.  The student makes drawings for the teacher. |
| Greetings  (Greetings between teacher and student in daily life) | (Not mentioned) | The student greets with teachers.  Teachers respond to the greetings from student. | (Not mentioned) | (Not mentioned) | The student greets with teacher. | Teacher says goodbye to the students one by one at the end of the year.  Teacher welcomes students at the beginning of the day. |
| Having an active interest in each other's life  (Every attempt of the teacher/student to get to know/understand the student/teacher) | (Not mentioned) | (Not mentioned) | (Not mentioned) | The student feels that the teacher is interested in their life.  Focusing not only on school but also on other aspects of the student's life.  Taking time to get to know the student so you can understand their behavior better when you're in conflict.  Showing interest in the students’ lives (e.g. seeing if they have a new book bag, if their hair has been cut,..). | (Not mentioned) | Teacher listens to students.  Teacher, at the beginning of the week, ask students what they did during the weekend.  Teacher understand his or her students by means of reactions to physical contact.  Teacher understands his or her students just observing them. |
| Having fun together  (Every act of teacher and students enjoying each other’s company and doing fun activities) | Teacher uses humour with his students (joking, light-hearted).  Teacher is cheerful towards pupils.  Balance between work and play.  Balance authority and playfulness.  Teacher is cheerful towards pupils. | The student has fun in class.  The student actively seeks out teachers for fun and games.  The student enjoys their teachers’ lessons.  Teacher jokes with students.  Teacher and student having fun together.  Teacher and student playing together after class.  The student is interested in courses and the teachers.  Teacher and student are happy together.  Fun between the teacher and the student. | The student is cheerful with the teacher.  The student likes the physical education hours because within it we play together.  The student smiles to the teacher.  Fun between teacher and student.  Teacher and student joke together.  Teacher and student laugh together.  Teacher does body percussion activities with students.  Teacher does frequent breaks using funny activities to keep students up.  Teacher feels students are happy.  Teacher lets students enjoy the school.  Teacher plays with students.  Teacher provides enjoyable activities.  Teachers provides funny elements in the lesson to improve the school climate. | Teacher interacts with the student in a playful and warm way. | Student/Teacher jokes with each other.  Teacher and student play games. | Teacher and student joke together.  Teacher tells stories to the student. |
| Interest in lessons  (Student is interested in what teacher teaches) | (Not mentioned) | The student is interested in what teacher teaches. | (Not mentioned) | (Not mentioned) | The student likes the teacher's lessons.  Student looks forward to attending the teacher's class. | The student enjoys spending time with the teacher. |
| Keeping track of the teacher  (The student keeps track of the teacher or expects presence of the teacher) | (Not mentioned) | (Not mentioned) | (Not mentioned) | The student chooses their teacher, prefer their teacher to another teacher | The student actively approaches teacher.  Students gather around the teacher. | The student is sad when teacher is leaving |
| Looking up to the teacher  (Teacher as a point of reference for students and in the position to model certain behaviors) | Acting as a role model for students.  The student looks up to teacher  Acting as a role model for  students as a blank page. | The student copies the behaviors of the teacher. | (Not mentioned) | Difference how students treat their teacher vs. their parents, e.g. they look up more to their teacher, students listen more to their teacher. | (Not mentioned) | The student sees teacher as a point of reference. |
| Open communication  (Every act of the teacher to open a channel of communication towards students and students sharing personal experiences/opinions) | The student is willing to share on experiences.  Balance between letting the student tell and starting the lessons.  Being a confidant. | The student and teacher feel at ease being together  The student and teachers open hearts to each other.  The student and teachers share what's going on in the classroom.  The student shares personal and private matters with the teacher.  Teachers actively communicate with the students.  Teachers and students have common topics to talk about.  Teachers expect feedback from students.  Teachers initiate chats with students.  Teachers respond to the greetings from students. | The student complains about homework to the teacher.  The student talks about personal and familiar things with the teacher.  The student talks less about his/her life as they grown.  Teacher can discuss more serious contents (like civic education).  Teacher helps students inside the lesson and the classroom context.  Teacher invites students to express their problems and negative emotions.  Teacher invites student to talk to his or her if they need.  Teacher provides feedback. | The student feels safe in the classroom (safe enough to say things, dares to share things with fellow pupils).  By talking about the conflict afterwards the student accepts more why the teacher was angry and they were punished.  Creating opportunities for the student to show how it feels (e.g. working with notes read by the teacher, working with colors to show how the student feels,..).  Explaining that as a teacher you don't want to get angry but that sometimes it is necessary.  Explaining why you're angry at the student e.g. because they didn't follow the rules.  Making it clear to the student that a conflict is only temporary and the teacher won't stay angry forever.  Remember things about the students even though they are no longer in your class to maintain the bond (e.g. ask how the dog is doing). | Student shares their joy with teachers.  Student talks to teachers about the little things in their lives.  Student shares their personal things with the teacher. | The student asks for being listened to.  The student communicates by means of their face expressions.  The student complains about homework to the teacher.  The student thanks the teacher because he or she permits them to talk about everything.  The student needs to and/or teacher permit express the student’s feelings and situations to the teacher.  The student says easily to teacher that they didn't complete their tasks or that they cannot complete it.  The student shares his/her difficulties with the teacher.  The student talks about joy and/or personal and familiar events to the teacher.  The student talks about personal and familiar events to the teacher.  Teacher communicates easily with students when anxious.  Teacher encourages students to share their doubts to all the classroom.  Teacher is available in order to let students express their emotions and personal situations.  Teacher lets students talk to him or her all together within the classroom.  Teacher schedules and provides a suitable spot within which students can talk about everything and be listened to.  Teacher brings students out of the classroom when he/she needs to talk to him or her. |
| Regarding as a member of the family/friend  (Student/teacher regards the teacher/student as his or her family/friend) | (Not mentioned) | The student regards the teacher mother. | (Not mentioned) | Forster. | The student calls the teacher mother.  Teacher and student communicate like friends. | The student calls the teacher Mother/ Grandma.  The student perceives the teacher and the classroom as a family.  The student talks to the teacher as she were part of their family.  Teacher feels as a friend of students when close to them.  Teacher feels part of students’ life.  Teacher perceives his or her students as a family. |
| Respect  (Teachers and students respect each other and/or the rules) | Balancing different cultures and having sufficient respect. | The student respects his/her teachers.  Teacher respects students.  The student is aware of that they need to be quiet when the teacher is present.  The student obeys teachers. | The student puts the hand up to talk with the teacher.  Respect.  The student doesn't act in a bad way because of their respect towards the teacher.  The student respects rules.  The student respects the role of the teacher.  Teacher approaches to students respecting them.  Teacher expects respect from students.  Teacher respects students individual differences.  Teacher respects students’ needs.  The student talks to the teacher one by one without overlapping and putting the hand up.  Teacher approaches to students respecting them. | Finding it important to have respect for each other even during conflict. | The student is willing to listen to the teacher's instructions. | The student doesn't want to make teacher angry. |
| Safe haven  (Student seeks comfort and reassurance from the teacher) | (Not mentioned) | (Not mentioned) | (Not mentioned) | The student is comforted by the teacher. | The student seeks comfort from the teacher.  Teacher comforts student when he is sad. | The student cries and goes close to the teacher to talk to him or her.  The student feels reassured by teacher or keep calm thank to the teacher.  The student needs to be reassured.  Teacher calms students if agitated.  Teacher comforts/cuddles the student.  Teacher cuddles students in order to provide them comfort.  Teacher is a psychological support for students. |
| Satisfactory relationship  (Student/teacher has positive feelings about relationship with teacher/student) | (Not mentioned) | (Not mentioned) | (Not mentioned) | The student shows that they like the teacher (e.g. by telling things, making drawings,..)  The student chooses their teacher, prefer their teacher to another teacher. | The student looks forward to meeting the teacher.  The student is very relaxed being with the teacher.  The student is happy to get attention from teacher.  The student is happy to be understood and affirmed by teacher.  The student is happy to get attention from teacher. | The student feels accepted and free with the teacher.  The student feels better with a teacher instead of another one.  The student wants to go to school only when there's that specific teacher.  Teacher feels emotionally involved in the relationship.  Teacher feels full of affection and positive vibes towards students.  Teacher feels self-efficacy providing affection to students.  Teacher is happy when he or she make students feel better.  Teacher is satisfied about his or her closeness towards students. |
| Secure base  (Student feels a sense of safety in their relationship with the teacher) | Creating a safe environment for the students.  Easy relationship.  Good relationship.  The student should feel accepted by the teacher  Interacting with the students in a warm and friendly way.  The student shows he/she likes the teacher.  Teacher and students get to know each other and what to expect from each other. | The student and teacher feel at ease being together.  The student and teacher get along well.  Teacher feels close to their students.  Teacher feels relieved.  Teacher treats students as friends.  Teacher treats students gently. | The student is safe with the teacher.  The student feels understood by the teacher.  The student perceives a good climate in the classroom.  The student perceives teacher wants to help them.  The student feels caring.  Teacher says students she feels good with them.  Teacher wants students feel good with him or her.  Teacher perceives feelings of sympathy or dislike towards students.  Teacher likes some students.  Teacher prefers to communicate and enter in a relationship with students he or she likes.  The student perceives a good climate in the classroom.  Teacher says students she feels good with them.  Teacher has a feeling of tenderness towards students. | The student feels good in the classroom, feels secure and accepted.  The student has the feeling that they can go to the teacher (e.g. for problems, questions, to say something). | The student feels safe together being with the teacher.  The student is not afraid of teachers.  The student is very relaxed being with the teacher.  The student doesn't hold grudges. | The student feels better with a teacher instead that another one (e.g., feel safe).  The student is safe with the teacher.  The student feels a lot of confidence towards the teacher.  Teacher feels a psychological safe base for students. |
| Seeking practical help  (Student feels free to seek help from the teacher or the teacher is available to help student when needed) | Being available to listen to the students. | (Not mentioned) | Teachers allows students to rely totally on her.  Teacher listens to students.  Teacher helps students inside the lesson and the classroom context  Teacher satisfies students’ needs | Let students know they can ask questions if they can't do something.  Helping students when needed but letting them try for themselves first. | The student will seek teacher helps when necessary.  Teacher provides extra help when the student has learning difficulties.  Teachers help students keep clean. | The student asks for advices/helps.  Teacher helps students during school work.  Teacher tries to solve students’ problems. |
| Trust  (Student and teacher having faith in one another) | Being a confidant.  Balancing between being a confidant and maintaining distance.  Student and teacher having faith in one another.  Mutual trust. | The student trusts teacher.  Teacher trusts his/her students.  Teacher and student trust each other.  Proximity (emotional). | The student trusts the teacher.  The student makes the teacher satisfied because they trust her.  The student trusts the teacher when he or she reproach or praise him.  Teacher perceives students trust him or her.  The student knows teacher can help them.  The student comes to teacher to solve discrepancies.  The student relies on the teacher.  Teachers allows students to rely totally on her. | The student trusts the teacher (e.g. trust the teacher enough to come to the teacher with problems).  Building trust with the student.  Mutual trust between pupil and teacher.  Younger students want to trust the teacher. | Mutual trust between pupil and teacher. | Teacher tries to build a trust relationship.  The student relies on the teacher. |

**Appendix 3 Cross-site Metatheme: Conceptualization and Manifestation of Conflict**

| Metathemes | Sites-specific Themes (1st round) | | | Sites-specific Themes (2nd round) | | |
| --- | --- | --- | --- | --- | --- | --- |
|  | Belgium | China | Italy | Belgium | China | Italy |
| Conflict resolution  (The teacher/student solves a conflict) | (Not mentioned) | (Not mentioned) | (Not mentioned) | How the teacher solves conflicts between the students themselves.  Discussing with the student how to avoid conflict in the future.  Apologizing to students for getting angry.  Working on repairing the relationship after a conflict e.g. asking how they feel afterwards, how they can make up for their behavior.  Not finding a solution for conflicts between students themselves. | Teacher lets students understand why they behaviors are not appropriate. | The student says -Sorry- to the teacher after a bad behavior.  Students, thanks to the teacher, understand that they had a bad behavior.  Teacher lets students understand and reflect about their bad behaviors.  Teacher tries to solve conflict talking to students. |
| Dissatisfaction and negative feelings  (The teacher/student not being content about his/her teacher/students, or the teacher/student implicitly expresses feelings of anger, hostility or disapproval towards the student/teacher) | The student does not share what is wrong. | The student does not like teachers.  The student doesn't care about teachers.  The student is afraid to share their thoughts to teachers.  The student is afraid of teachers.  The student feels teachers are mean. | The student doesn't trust the teacher.  The student doesn't believe what teacher says.  The student doesn't want to do what teacher asks them to do.  The student understands when teacher is not sincere.  The student thinks teachers is just an adult to be satisfied.  The student criticizes what teacher says and proposes. | The student gives an angry look to the teacher.  The student becomes angry when the teacher tells them they're not following the rules or doing something wrong.  Teacher getting angry when a student hurts other students.  Teacher is more disappointed than angry when there is conflict. | The student feels aggrieved and cry because of what the teacher did.  The student does not have direct conflicts with teacher.  The student and teacher are sulking.  Teacher is angry with student.  The student uses expressions to express dissatisfaction with the teacher. | The student is angry and avoidant when teacher doesn't satisfy their need of dependency.  The student is sad within a conflict.  The student feels humbled by teacher.  The student is angry towards the teacher.  The student is fine when teacher rises the voice against them.  The student snorts and pouts at the teacher.  Teacher is angry towards students because they didn't complete their tasks.  The student protests because teacher is angry.  Teacher is angry towards the student.  Teacher is angry when a student is behaving bad towards other students.  Teacher perceives his or her face expression is changing when he or she is angry. |
| Doubting (students don’t trust teachers) | The student does not share what is wrong.  The student is more aloof towards the teacher. | The student doesn’t care about teachers. | The student criticizes what the teacher says and proposes.  The student wonders if they're interested in what they're doing at school.  The student wonders if they're learning something through the teacher.  The student doesn't trust the teacher.  The student doesn't believe what the teacher says. | (Not mentioned) | The student does not trust the teacher.  The teacher does not trust the student. | (Not mentioned) |
| Fear  (Student is afraid of teacher because the teacher is harsh or strict) | (Not mentioned) | (Not mentioned) | (Not mentioned) | (Not mentioned) | The student is afraid of teacher. | The student is afraid of the teacher.  The student doesn’t go to school because they're afraid of the teacher. |
| Non-communication  (Student/teacher does not to communicate with the teacher/student) | (Not mentioned) | (Not mentioned) | (Not mentioned) | Having conflict with a student because they don't listen to you. | The student and teacher ignore each other out of dislike.  The student deliberately does not speak when asked by the teacher.  The student refuses to communicate with teachers. | Teacher feels some student find it difficult to talk or be close to him or her.  The student doesn't want to talk with the teacher. |
| Non-conflict  (The teacher perceives no conflict with the student) | (Not mentioned) | (Not mentioned) | (Not mentioned) | Not having many very difficult conflicts. | The teacher believes there has been no conflict with the student. | Teacher doesn't feel the conflict.  Teacher doesn't feel conflicts because students are too small.  Teacher doesn't reproach students if they do something wrong.  The student doesn't behave badly towards the teacher.  Teacher doesn't reproach students if they do something wrong. |
| Non-cooperative behaviors  (students’ actions or behaviors that are not collaborative, cooperative, or helpful in achieving a goal or task) | (Not mentioned) | (Not mentioned) | (Not mentioned) | The student answers rudely.  The student doesn't do what the teacher asks.  The student does something that is against the rules.  The student is very defensive towards the teacher in a conflict e.g. by saying the teacher is always angry at them.  The student shows irritating behavior that disrupts the lessons.  The student pushes at the boundaries of the rules e.g. testing to see what they can get away with.  The student shows annoying behavior because they are bored in class or because they can't follow the lesson e.g. trash, don't participate anymore,... (The student shows irritating behavior when they're bored in class or don't understand the lesson).  Irritating behavior by the student leads to conflict with the teacher. | The student being unreasonable and make a scene to teacher.  The student resists teacher criticism.  The student has complaints about teachers.  The student provokes teacher.  The student is arguing with teachers. | The student acts in a bad way because they are looking for help or demanding attention.  The student is naughty.  The student continues acting in a bad way even after reproaches.  The student exaggerates with their behavior.  The student doesn't do what teacher ask them to do.  The student exaggerates with their behavior.  The student has a provocative attitude towards the teacher.  The student kicks the teacher.  The student teases the teacher. |
| Punishment  (The teacher uses corporal punishment) | (Not mentioned) | (Not mentioned) | (Not mentioned) | (Not mentioned) | Teacher physically punishes student. | Teacher confiscate students’ diary because they're acting in a bad way.  Teacher removes students from a game. |
| Reproachments  (The teacher reproaches students) | (Not mentioned) | Teacher criticizes pupils, pupils rebel. | (Not mentioned) | Reacting when a student repeatedly shows irritating behavior or behavior that is against the rules e.g. commenting on the behavior.  Accidentally giving a comment to the wrong student who had done nothing wrong. | Teacher considers the psychological needs of student and reproach them in a positive way. | The student feels bad when reproached.  Teacher is fine when he or she reproaches students.  Teacher is not fine reproaching students because he or she feel that, as teacher, is behaving in a different way.  Teacher needs to reproach students to let them respect rules.  Teacher reproaches students.  Teacher reproaches students rising the voice.  Teacher reproaches students just looking at them.  Teacher reproaches students because other strategies to correct their behaviors are inefficient/they're doing other things during the lesson/they're not autonomous/they've a bad behavior among each other.  Teacher reproaches students when they do something dangerous/when they don't respect the nature/when they're exaggerating within their behaviors. |
| Struggling relationship  (Teacher feels struggled with the student) | Interaction with the student is more difficult, less smooth and easy. | Difficult relationship for teachers and students.  Teacher doesn't like students.  Teacher criticizes pupils, pupils rebel. | Teacher is angry about students.  Teacher dislikes some students.  Teacher perceives feelings of sympathy or dislike towards students.  Teacher is angry about students. | Finding ways for a student to express themselves including during a conflict e.g. a notebook they can give the teacher.  Teacher raises their voice if the class or a student doesn't listen.  The student keeps discussing the conflict and talking about his side of the conflict.  Conflict weighs on the teacher.  Difficult to teach when there is conflict with a student.  When teacher has to react in front of the whole class e.g. raising their voice also emphasizing that there were some students who were good). | The student does not obey the teacher's instructions. | Teacher enters in conflict towards students because they disturb the climate of the classroom/students laugh during the lesson/he or she is nervous/he or she is tired or stressed/students presenting difficulties dealing their angry/ students that don't respect rules/ when he or she has higher expectation towards them.  Teacher feels animosity towards students.  Teacher feels bad when he or she is not able to help students.  Teacher finds difficulties dealing with conflicts.  Teacher is angry because he or she doesn't feel understood by students.  Teacher is demoralized when he or she doesn't understand students.  Teacher needs to physically block students with bad behavior.  Teacher realizes he or she is not behaving good during a conflict. |
| Unfairness  (The teacher treats the student unfairly) | (Not mentioned) | (Not mentioned) | (Not mentioned) | The student shows that they feel they were treated unfairly e.g. by telling their parents.  The student feels unfairly treated when the teacher compliments another student. | Teacher is not fair to student. | The student feels treated worse than other classmates.  The student, when reproached, considers it unfair. |

**Appendix 4 Cross-site Metatheme: Conceptualization and Manifestation of Dependency**

| Metathemes | Sites-specific Themes (1st round) | | | Sites-specific Themes (2nd round) | | |
| --- | --- | --- | --- | --- | --- | --- |
|  | Belgium | China | Italy | Belgium | China | Italy |
| Asking for help constantly  (The student keeps looking for help, even when it is not really needed. ) | (Not mentioned) | (Not mentioned) | (Not mentioned) | The student asks for help when it is not really needed. | The student still relies on teacher to complete tasks he could do on their own.  The student still turns to teachers when faced with minor issues that don't require help.  The student still turns to teachers when faced with unnecessary help with life's chores. | The student is dependent because they're convinced teacher can do everything for them.  The student asks for help to complete their tasks.  The student calls the teacher asking for help in a private way. |
| Confirmation seeking  (The student looks for confirmation by the teacher) | (Not mentioned) | (Not mentioned) | (Not mentioned) | Asking a lot of questions of the teacher and seeking validation e.g. do you think I did a good job).  The student is dependent on the teacher due to insecurities. | The student needs direct teacher supervision to complete tasks. | The student needs constant confirmation. |
| Demanding attention  (The student demands attention or teacher improves students’ getting attention) |  |  |  | The student seeks attention from the teacher to compensate for attention it doesn't receive at home.  Constantly wanting to tell things to the teacher e.g. when she is a little bit hurt or during lunch.  Ignoring the student when it cries because they believe that giving it attention will result in continued crying and lack of effort.  Being loud in class to get attention from the teacher. | The student wants to get the teacher's attention. | The student calls the teacher.  The student puts the hand up to call the teacher.  The student cries in order to get teacher's attention.  The student demands attention.  The student disrupts the classroom climate if the teacher doesn't fulfill their need for dependency. |
| Emotional dependency  (chronically and excessively seeking proximity and support) | (Not mentioned) | (Not mentioned) | (Not mentioned) | When you offer students more help and presence they're more likely to choose that than working independently.  Some students seek the teacher out because they don't know what to do during free play time. | The student expects the teacher to meet all his needs.  The student doesn't want teacher to leave.  The student sees teacher as parent.  The student is unhappy because the teacher is close to other students.  The student does not sort out the boundaries with the teacher. | The student is sad when the teacher doesn't satisfy their need of dependency.  The student cries when teacher don't satisfy their need of dependency.  The student doesn't want to go to school if there's not the teacher.  The student feels anxiety when left alone.  The student interprets the need for autonomy as a rejection from the teacher.  The student pretends teacher should be available only for them.  If teacher always satisfies students’ need of dependency, students take advantage of that.  Some students become accustomed to seeking dependence.  Some students remain dependent to the teacher even when other classmates achieve independence.  Teacher feels essential for students, even if actually they don't need to be dependent on teacher. |
| Encourage students’ independence  (Teacher trying to make students more independent, and/or students are happy being independence) | Balance between wanting to help and maintaining distance. | Teacher encourages students to deal with problems independently.  Teacher encourages and accept students to have their own ideas. | When parents try to help students with homework, students reply that they can do it alone thanks to the teacher.  Teacher makes students independent.  Teacher makes students responsible of themselves  Scaffolding  When parents try to help students with homework, students reply that they can them alone thank to the teacher. | Block signs of dependency e.g. saying you can do it).  Helping the student find friends so it is less dependent on the teacher.  Giving students the tools to solve problems by themselves e.g. telling them how to close their backpack.  Having students work together and try solving problems together before going to the teacher.  Offering success experiences to students.  Reducing the amount of hugs or other signs of dependency e.g. by saying students can only give one hug per day.  Telling parents what the student needs help with so they can practice at home and the student can become more independent.  Thinking that it is important too make the student more independent e.g. by giving them positive feedback. | (Not mentioned) | The student feels better when he/she achieves a little bit more of independence.  The student feels better when they achieve a little bit more of independence.  Teacher assigns specific tasks in order to let students achieve more autonomy and independence.  Teacher encourages students to act alone.  Teacher feels inefficient trying to let students independent.  Teacher in a first moment satisfies students’ need of dependency and gradually he or she let them achieve more autonomy.  Teacher lets students achieve more self-esteem and self-security.  Teacher looks for a balance among dependency's satisfying and independence achievement.  Teacher provides stamps on students’ notebook as positive feedback to let them become independent.  Teacher reproaches students because they're not autonomous.  Teacher says students that they must be autonomous.  Teacher tries to explain students that other students need him or her as well.  Teacher tries to separate a student by him or her placing him next to another student or other students.  Teacher tries to let students be independent. |
| Instrumental dependency  (Support and help seeking in effective ways) | (Not mentioned) | (Not mentioned) | (Not mentioned) | Seeing dependency as the students not being able to function if the teacher were to be replaced by someone or to leave.  The student is dependent on the teacher when it comes to needing help with lessons. | The student has unreserved trust in teacher. | Teacher helps students if they have difficulties.  The student needs the teacher because they're not able to go to the bathroom alone.  The student needs the teacher to tie their shoes. |
| Non-overreliance  (The teacher does not perceive the student’s dependency) | (Not mentioned) | (Not mentioned) | (Not mentioned) | (Not mentioned) | Teacher did not find over-reliance. | Teacher doesn't feel dependence on students. |
| Physical closeness (inappropriately)  (The student needs to be constantly, excessively, or inappropriately physically closed to the teacher) | (Not mentioned) | (Not mentioned) | (Not mentioned) | The student constantly wants to be near the teacher.  The student gives hugs during the lessons and teacher doesn't think it's appropriate e.g. because she is teaching a class).  The student comes to give hugs or show other signs of dependency.  Not being able to start before other students start. | The student doesn't want the teacher to leave.  The student hanging around the teacher. | The student comes to the teacher's desk.  The student constantly asks teacher to come towards them.  The student holds teachers' hands.  The student needs constantly the physical presence of the teacher.  The student needs to come to the desk of the teacher.  The student prefers to stay with the teacher instead of the classmates.  The student stays in teacher's lap.  The student wants to stay attached to the teacher during breaktime instead of playing with other students.  The student wants to stay physically close to the teacher. |
| Teacher improves students’ dependency  (Teacher’s behaviors in order to not make students independent) | (Not mentioned) | (Not mentioned) | (Not mentioned) | (Not mentioned) | (Not mentioned) | Teacher asks students if they need help.  Teacher satisfies students’ need of dependency.  Teacher thinks sometimes students are dependent by teachers because teachers want them dependent. |

**Appendix 5 Cross-site Metatheme: Conceptualization and Manifestation of Other Dimensions**

| Metathemes | Sites-specific Themes (1st round) | | | Sites-specific Themes (2nd round) | | |
| --- | --- | --- | --- | --- | --- | --- |
|  | Belgium | China | Italy | Belgium | China | Italy |
| Authority  (Teacher acts as an authoritative figure) | Being fair and strict.  Balance authority and playfulness.  Balancing authority and being a warm teacher.  Teacher explains what students can and cannot do. | Teachers are strict with their students.  The student is in awe of their teachers.  The student is aware of that they need to be quiet when the teacher is present.  The agreement between teacher and student.  The student obeys teachers. | Authority. | (Not mentioned) | The student has unreserved trust in teacher. | Teacher must be authoritative and not permit conflict or lack of respect. |
| Balance  (Teacher needs to keep balance in the classroom) | Balancing authority and being a warm teacher.  Balancing between being a confidant and maintaining distance.  Balance authority and playfulness.  Balance between letting the student tell and starting the lessons.  Balance between wanting to help and maintaining distance.  Balance between work and play. | (Not mentioned) | (Not mentioned) | Finding a balance between helping and consoling a student but also not letting the student become too dependent.  Finding balance between being strict when a student doesn't listen and trying to understand the reasons for the behavior behind the conflict.  Allowing some physical signs of dependency such as hugs as long as it is within limits. | (Not mentioned) | (Not mentioned) |
| Distance  (The coolness or unfriendliness in the way that teacher/student behaves toward each other) | (Not mentioned) | (Not mentioned) | (Not mentioned) | Feeling like you don't have a good relationship with a student e.g. they never come to tell you something.  The student shows no emotion or reaction when a teacher asks them about what happened or why a conflict happened. | The student is not attracted to the teacher.  The student is reluctant to invite teachers to participate in chats.  The student is reluctant to reveal their hearts when teachers communicate with them.  The student does not initiate conversation with teacher.  The student does not greet with teacher.  The student does not like the teacher's class.  The student doesn't care about teacher.  The student doesn't care about teacher’s criticism.  The student behaves restrainedly when the teacher was present.  The student has a sense of distance from the teacher.  The teacher expresses to the student that he does not like him. | The student is clever and they manifest affection because they want something in exchange.  Teacher doesn't feel closeness towards students.  Teacher enters in conflict towards students because students don't permit teacher to establish a relation.  Teacher feels uncomfortable when students talk about their personal events because maybe parents don't want their students talk about their personal events.  Teacher prefers students don't talk about their private issues.  Teacher needs to understand if students manifest affection naturally or if they want something in exchange. |
| Fairness  (Teacher treats students in a fair way) | (Not mentioned) | (Not mentioned) | (Not mentioned) | Trying not to punish a student unjustly, not thinking they've been bad just because they have been in the past. | Teacher is fair to students. | Teacher wants to be fair towards all the students.  Teacher doesn't feel conflict because he or she tries to be as fair as possible. |
| Increasing student's motivation (Teacher improves students) | (Not mentioned) | Teachers say motivational words to students.  Teachers acknowledge students' strengths.  Teachers give students gifts to motivate them.  Teachers motivate students by comparing different students. | When parents try to help students with homework, students reply that they can them alone thank sto the teacher.  Teacher tries to understand in which way students can learn better.  Teacher let students understand their emotions.  Teacher criticizes students in a positive way, because he or she doesn't want to judge them but he or she cares of them and wants they can improve themselves. | Having a reward system where students go up a point when they follow the rules or lose a point when they don't.  Making the student feel good, emphasizing the positive and strengths of the student and working on self-confidence (e.g. by giving compliments, positive feedback, ..).  Rewarding good behavior instead of always focusing on the bad motivates the student and reduces conflict. | Teacher gives small gifts to students | Teacher says that students they are good. |
| Patience  (Teacher stays calm and do not get annoyed by the student) | Teacher is patient with students. | Teachers are patient with students. | Teacher waits for the individual time of students.  Teacher is patient with students. | When the student goes into conflict the teacher doesn't get angry so the conflict doesn't escalate.  As a teacher, being able to tolerate a lot when it comes to conflict or students not listening. | (Not mentioned) | Teacher always maintains a quiet tone of voice. |
| Strictness  (Teacher regards many actions of students as unacceptable and do not allow them) | Being fair and strict.  Teacher explains what students can and cannot do. | Teachers are strict with their students.  Students are under pressure.  Teachers criticize students without affecting the teacher-student relationship.  Teacher criticizes pupils, pupils rebel. | Teacher criticizes students in a positive way, because he or she doesn't want to judge them but he or she cares of them and wants they can improve themselves. | Being firm and strict when it comes to the rules in the class and during conflict. | Teacher is strict. | Teacher is strict towards students. |
